# CXCR4 hyperactivation cooperates with TCL1 in CLL development and aggressiveness

**DOI:** 10.1038/s41375-021-01376-1

**Published:** 2021-08-06

**Authors:** Richard Lewis, H. Carlo Maurer, Nikita Singh, Irene Gonzalez-Menendez, Matthias Wirth, Markus Schick, Le Zhang, Konstandina Isaakidis, Anna Katharina Scherger, Veronika Schulze, Junyan Lu, Thorsten Zenz, Katja Steiger, Roland Rad, Leticia Quintanilla-Martinez, Marion Espeli, Karl Balabanian, Ulrich Keller, Stefan Habringer

**Affiliations:** 1 Department of Hematology, Oncology and Cancer Immunology, Campus Benjamin Franklin, Charité - Universitätsmedizin Berlin, corporate member of Freie Universität Berlin and Humboldt-Universität zu Berlin, Berlin, Germany; 2 School of Medicine, Technische Universität München, Munich, Germany; 3 Internal Medicine II, School of Medicine, Technische Universität München, Munich, Germany; 4 Institute of Pathology and Neuropathology and Comprehensive Cancer Center Tübingen, Eberhard Karls Universität Tübingen, Tübingen, Germany; 5 European Molecular Biology Laboratory (EMBL), Heidelberg, Germany; 6 Department of Medical Oncology and Hematology, Universitätsspital and Universität Zürich, Zurich, Switzerland; 7 Institute of Pathology, Technische Universität München, Munich, Germany; 8 TranslaTUM, Center for Translational Cancer Research, Technische Universität München, Munich, Germany; 9 Institute of Molecular Oncology and Functional Genomics, TUM School of Medicine, Technische Universität München, Munich, Germany; 10 German Cancer Consortium (DKTK), German Cancer Research Center (DKFZ), Heidelberg, Germany; 11 Université de Paris, Institut de Recherche Saint-Louis, EMiLy, INSERM U1160, Paris, France; 12 CNRS, GDR3697 “Microenvironment of Tumor Niches”, Micronit, France; 13 OPALE Carnot Institute, The Organization for Partnerships in Leukemia, Hôpital Saint-Louis, Paris, France; 14 Max-Delbrück-Centrum für Molekulare Medizin, Berlin, Germany; 15 Berlin Institute of Health at Charité (BIH), Berlin, Germany

**Keywords:** B-cell lymphoma, Oncogenesis, Cancer models, B-cell lymphoma, Oncogenes

## Abstract

Aberrant CXCR4 activity has been implicated in lymphoma pathogenesis, disease progression, and resistance to therapies. Using a mouse model with a gain-of-function *CXCR4* mutation (*CXCR4*^*C1013G*^) that hyperactivates CXCR4 signaling, we identified CXCR4 as a crucial activator of multiple key oncogenic pathways. CXCR4 hyperactivation resulted in an expansion of transitional B1 lymphocytes, which represent the precursors of chronic lymphocytic leukemia (CLL). Indeed, CXCR4 hyperactivation led to a significant acceleration of disease onset and a more aggressive phenotype in the murine *Eµ-TCL1* CLL model. Hyperactivated CXCR4 signaling cooperated with TCL1 to cause a distinct oncogenic transcriptional program in B cells, characterized by PLK1/FOXM1-associated pathways. In accordance, *Eµ-TCL1;CXCR4*^*C1013G*^ B cells enriched a transcriptional signature from patients with Richter’s syndrome, an aggressive transformation of CLL. Notably, *MYC* activation in aggressive lymphoma was associated with increased CXCR4 expression. In line with this finding, additional hyperactive CXCR4 signaling in the *Eµ-Myc* mouse, a model of aggressive B-cell cancer, did not impact survival. In summary, we here identify CXCR4 hyperactivation as a co-driver of an aggressive lymphoma phenotype.

## Introduction

CXCR4 is a G-protein-coupled receptor regulating hematopoietic stem cell homeostasis, myelopoiesis, lymphopoiesis, and homing of immune cells toward its ligand C-X-C motif chemokine 12 (CXCL12) [1–3]. CXCL12 binding induces a multitude of G-protein-dependent and -independent signaling pathways including PI3K/AKT, MAPK/ERK, and PLC/Ca^2+^ signaling [4]. CXCR4 is phosphorylated at the C-terminus and rapidly internalized after binding of CXCL12 [5]. Truncating mutations affecting the C-terminus lead to increased activity of CXCR4 signaling in response to its ligand by impairing receptor desensitization and internalization without impairing receptor expression level [6–8], which has been modeled in mice [9].

B cells in particular are highly dependent on the interaction of CXCR4 and its ligand CXCL12 at multiple stages during the germinal center reaction [10]. Molecularly targeted imaging studies have revealed enhanced CXCR4 expression in various B-cell non-Hodgkin lymphomas [11–13]. CXCR4 is of particular interest in chronic lymphocytic leukemia (CLL) and diffuse large B-cell lymphoma (DLBCL), having been associated with adverse prognosis in both diseases [14, 15]. CXCR4 is overexpressed in CLL patients and involved in interactions of CLL cells with their microenvironment, specifically the protection from apoptosis by the provided ligand CXCL12 [16–18]. In DLBCL, CXCR4 expression correlates with bone marrow infiltration [19] and has been implied in mediating resistance to B-cell receptor and PI3K inhibitors [20].

Genetically engineered mouse models for CLL and aggressive B-cell lymphoma, specifically the *Eµ-TCL1* mouse model of CLL [21] and the *Eµ-Myc* mouse model of aggressive MYC-induced B-cell lymphoma [22], are essential for studying leukemia/lymphoma pathogenesis and complex biological systems like chemokine receptor pathways in B-cell pathobiology. The *Eµ-TCL1* mouse, in which B-cell-directed TCL1 expression drives development of a CLL-like disease, is the most commonly used model for high-risk CLL and has been extensively used for mechanistically assessing oncogenes and tumor suppressors in CLL [23, 24]. By using the *Eµ-TCL1* model, Chen et al. showed that downregulation of surface CXCR4 expression and inhibition of CXCR4 downstream signaling in CLL cells can be observed upon treatment with the clinically approved Bruton’s tyrosine kinase (BTK) inhibitor ibrutinib [25]. CXCR4 inhibitors are currently under investigation in clinical trials in CLL [26].

Mutations of *CXCR4* are present in DLBCL patients, but no functional studies have been performed to further inquire their function in this disease [27, 28]. Intriguingly, rare germline mutations in *CXCR4* have been found in CLL patients with familial clustering [29] and mutations in regulatory regions of *CXCR4* were discovered in biopsies of CLL with aggressive transformation (Richter’s syndrome) [30]. It is however not resolved if and how they affect CXCR4 pathway activity and disease progression. Despite a plethora of evidence linking CXCR4 to the pathogenesis of CLL and DLBCL, it is unknown if and how enhancing CXCR4 pathway activity can alter the course of B-cell lymphoproliferation, and B-cell leukemia/ lymphoma development and progression.

In this study, we employed the *Eµ-TCL1* and *Eµ-Myc* mouse models to interrogate the role of hyperactivated CXCR4 signaling in B-cell lymphoproliferation and B-cell leukemia/lymphoma pathogenesis. CXCR4 hyperactivation was achieved by a mouse model harboring *CXCR4*^*C1013G*^, a mutation resulting in a truncated C-terminus missing phosphorylation sites for CXCR4 internalization and consequently enhanced downstream signaling [9]. This mutation is characteristic for WHIM-syndrome (warts, hypogammaglobulinemia, infections, myelokathexis) patients and allows the investigation of CXCR4 hyperactivation on B-cell lymphoproliferation and lymphoma development.

## Material and methods

### Animal experiments

Genotyping was performed as previously described [9, 21, 31]. All analyses included heterozygous female and male animals on a C57BL/6J background. Animal caretakers, but not researchers performing experiments, were blinded for genotypes. Mice were allocated to groups based on genotype, thus no randomization was performed. All animal experiments were performed in accordance with Federation of European Laboratory Animal Science Associations guidelines and with permission of the respective authorities (Regierung von Oberbayern, Munich, Germany & Landesamt für Gesundheit und Soziales, Berlin, Germany).

### Flow cytometry

Staining was performed in PBS (ThermoFisher Scientific, Waltham, MA) containing 0.5% BSA (Carl Roth GmbH, Karlsruhe, Germany). To distinguish live from dead, cells were stained with PI, DAPI, or Invitrogen Fixable Aqua Dead Cell Stain Kit (ThermoFisher Scientific, Waltham, MA). Data analysis was done with FlowJo™ Version 10.6.0 (FlowJo, Ashland, OR). An extensive list of all fluorescently labeled antibodies can be found in Supplementary Table T1. Gating strategies are depicted in Supplementary Fig. S1.

### Histopathological analysis

Tissue samples were fixed in 4% formaldehyde for 48 h, paraffin embedded, sectioned, and stained using H&E. Samples of bone marrow were decalcified by incubating for 14 days with Osteosoft® (Sigma-Aldrich, Saint Louis, MI) after fixation.

### Immunohistochemistry

Immunohistochemistry on murine tissues was performed on an automated immunostainer (Ventana Medical Systems Inc., Oro Valley, AZ) according to the company’s protocols for open procedures with slight modifications. Appropriate positive and negative controls were used to confirm the adequacy of the staining. The histologic samples were analyzed by an experienced pathologist (L. Quintanilla-Martinez). Photomicrographic images were acquired with an Axioskop 2 *plus* Zeiss microscope equipped with a Jenoptik (Laser Optik System, Jena, Germany) ProgRes C10 *plus* camera and software. Objectives Plan-Neofluar used were as follows: 1.25/0,035, 2.5×/0.075, 10×/0.30, 20×/0.50, and 40×/0.75. Final image preparation was performed with Adobe Photoshop CS6 (Adobe Inc., San José, CA).

### RNA sequencing

B cells from spleen and bone marrow of *WT, CXCR4*^*C1013G*^*, Eµ-TCL1*, and *Eµ-TCL1;CXCR4*^*C1013G*^ were isolated with CD19 directed magnetic beads (Miltenyi Biotec, Bergisch Gladbach, Germany). Only RNA samples with an RNA integrity number (RIN) > 7 were used for RNA sequencing. RIN was determined using Agilent RNA 6000 Nano Kit (# 5067-1513, Agilent, Santa Clara, CA) and the Agilent 2100 Expert software (version B.02.10.SI764). Library preparation and single-end sequencing was subsequently performed by Novogene (UK) (Cambridge, UK) on a HiSeq2500 (Illumina, San Diego, CA) with a sequencing depth of more than 20 M reads/sample. Fastq files were subsequently mapped to the murine reference genome GRCm38 with STAR.

Reads and transcripts per million (TPM) were estimated for each transcript using the transcript sequences from the GENCODE Release 25 (GRCm38) and the Salmon software (v1.3.0). Counts and TPM were summarized at the gene level by summing up the transcript values for each corresponding gene. Bioinformatic analyses are described in the Supplementary methods section.

### Immunoblot analysis

Protein extracts were prepared by incubating cell pellets in lysis buffer (50 mM Hepes, 150 mM NaCl, 1 mM EDTA, 2.5 mM EGTA and 0.1% Tween) supplemented with NaF, PMSF, and NaVO_4_ followed by sonification. Protein lysates were fractioned on SDS PAGE gels, transferred to Immobilon-P (Millipore, Burlington, MA) membranes and incubated with specific antibodies, and developed with Chemostar PC ECL & Fluorescence Imager (Intas Science Imaging, Göttingen, Germany). A list of antibodies used can be found in Supplementary Table T1. Quantification of protein expression levels was performed using ImageJ software (https://imagej.nih.gov/ij).

### Migration assay

Splenocytes were resuspended in RPMI-medium with or without 1 µM AMD3100 (Sigma-Aldrich, Saint Louis, MI). Cells were then loaded onto a Corning® Transwell® 96-well plate with 5 µm pore size. (Corning Inc., Corning, NY) with the other side of the membrane containing medium with/without 50 nM of CXCL12 (Peprotech, Rocky Hill, CT). Transwell® plates were incubated for 4 h at 37 °C. Migration of cells to the lower wells was measured using CountBright™ Absolute Counting Beads (ThermoFisher Scientific, Waltham, MA).

### Statistics

Statistical analyses were performed using GraphPad Prism Version 9.0. (GraphPad Software, La Jolla, CA). Error bars represent standard deviation. Bar graphs represent the mean. In animal experiments, a single data point represents an individual mouse. No sample size calculations were performed. Data from at least three mice per group were reported. In survival analyses, mice were excluded when the cause of death was not transgene-related (e.g. fighting, birth complications). A two-tailed Student’s *t* test was used to compare quantitative data between 2 independent samples. When comparing three or more groups, a one-way ANOVA with Tukey correction for multiple comparisons was used to compare group means. Survival data were compared using a logrank (Mantel–Cox) test. Results with a *P* value of less than 0.05 were considered significant.

## Results

### CXCR4 hyperactivation promotes a distinct transcriptional signature in non-malignant B cells

**Fig. 1:**
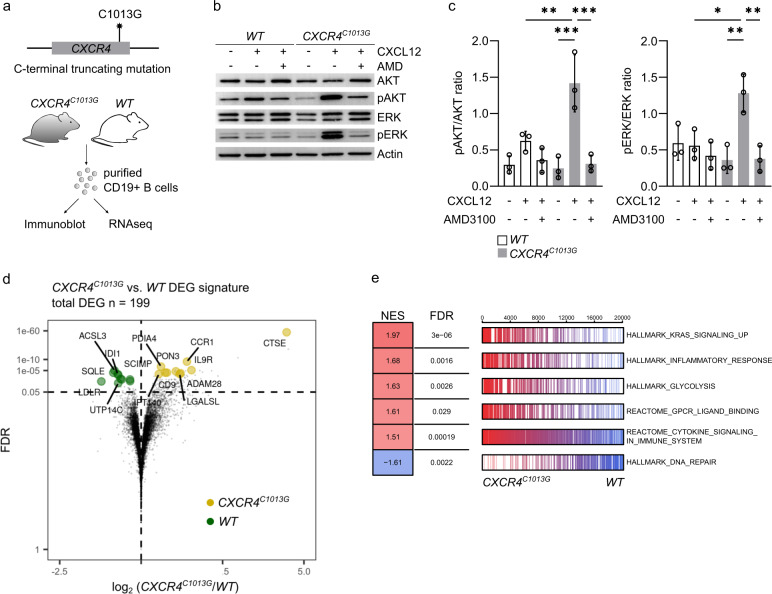
Identification of a transcriptional signature of hyperactivated CXCR4 signaling. **a** Illustration of the *CXCR4*^*C1013G*^ truncating mutation and outline of experimental setup for immunoblotting and RNA sequencing of splenic and bone marrow B cells. **b** Representative image of immunoblotting for AKT, pAKT (Ser473), ERK, pERK (Thr202/Tyr204), and Actin of CD19+ B cells treated with/without 50 nM CXCL12 and/or 10 µM CXCR4 inhibitor AMD3100 (AMD) (*WT*, *n* = 3; *CXCR4*^*C1013G*^, *n* = 3). **c** Relative pAKT to AKT and pERK to ERK protein expression by immunoblotting of *WT* and *CXCR4*^*C1013G*^ CD19+ B cells treated with/without 50 nM CXCL12 and/or 10 µM CXCR4 inhibitor AMD3100 (*WT*, n = 3; *CXCR4*^*C1013G*^, *n* = 3). **d** Volcano plot showing differentially expressed genes (DEG) of *CXCR4*^*C1013G*^ vs. *WT* CD19+ B cells (*WT*, *n* = 5; *CXCR4*^*C1013G*^, n = 5). **e** Gene set enrichment analysis (GSEA) of *CXCR4*^*C1013G*^ vs. *WT* CD19+ B cells showing normalized enrichment scores (NES) and false discovery rates (FDR) for curated gene sets listed in MsigDB [63, 64] of *CXCR4*^*C1013G*^ compared to *WT* CD19+ B cells. Statistical analyses were performed with one-way ANOVA with Tukey correction for multiple comparisons, *P < 0.05, **P < 0.01, ***P < 0.001. Error bars indicate standard deviation (SD).

To investigate the consequences of hyperactivated CXCR4 on intrinsic signaling in B cells, we purified CD19+ B cells from *CXCR4*^*+/C1013G*^ mice (*CXCR4*^*C1013G*^ from here on) and wild-type control mice (*WT* from here on) (Fig. 1a). To confirm enhanced CXCR4 signaling activity in *CXCR4*^*C1013G*^ B cells, we performed immunoblotting for phosphorylation of the well-established downstream effector kinases ERK and AKT. As expected, both ERK and AKT phosphorylation were readily increased upon stimulation with CXCL12 compared to control B cells (Fig. 1b, c). To get a more comprehensive understanding to which extent CXCR4 hyperactivation shapes the transcriptional landscape, we performed whole-transcriptome profiling of CD19+ B cells from *CXCR4*^*C1013G*^ and *WT* mice. *CXCR4*^*C1013G*^ B cells exhibited a set of 199 differentially expressed genes (DEGs, protein coding, *p*_adj_ < 0.05; logFC > 0.5 or <−0.5) compared to *WT* controls (Fig. 1d). Among upregulated genes in B cells with hyperactivated CXCR4 signaling, we identified genes involved in chemokine signaling, migration and adhesion (*Ccr1*, *Cxcl1*, *Cxcl2*, *Igfn1*, *Cntn2*, *Jaml*), NOTCH signaling (*Sorbs2*), inflammation, and cytokine signaling (*Il9r*, *Il7r*, *Csf2rb*, *Trem1*), B-cell maturation (*Rag1*, *Rag2*), plasma cell differentiation and proliferation (*Prdm1*), metabolism (*Pdk1*) and cell cycle progression (*Nek6*). Of note, the CXCL12-binding receptor *Ackr3* (encoding CXCR7), which is known to form heterodimers with CXCR4 and is dysregulated in inflammatory diseases and cancers [32], was upregulated. From these significantly regulated 199 DEGs in *CXCR4*^*C1013G*^ vs. *WT*, we generated a gene set defining the transcriptional profile of hyperactivated CXCR4 signaling (CXCR4a) in B cells (Supplementary Table T2). Subsequently, to test if CXCR4a represents a meaningful biological readout for enhanced CXCR4 signaling, we performed GSEA analysis with well-established published transcriptional signatures. We found that B cells of *CXCR4*^*C1013G*^ mice enriched chemokine receptor signaling, inflammatory response and cytokine signaling pathways (Fig. 1e). Fully in line with the implication of CXCR4 in B-cell cancers, we found cancer-relevant pathways such as Kras signaling and glycolysis as well as depletion of DNA repair pathways in *CXCR4*^*C1013G*^ B cells (Fig. 1e).

Thus, we here defined the transcriptional signature of hyperactivated CXCR4 signaling in B cells. Our data demonstrate the complexity of the transcriptional consequences of CXCR4 activation and represent a tool to assess activity of the CXCR4 pathway. Moreover, we found that enhancing CXCR4 signaling activity leads to the enrichment of several cancer-associated pathways, revealing that hyperactivated CXCR4 signaling might thereby predispose B cells for malignant transformation.

### Enhanced CXCR4 signaling cooperatively accelerates lymphoproliferation and promotes disease aggressiveness with TCL1

**Fig. 2:**
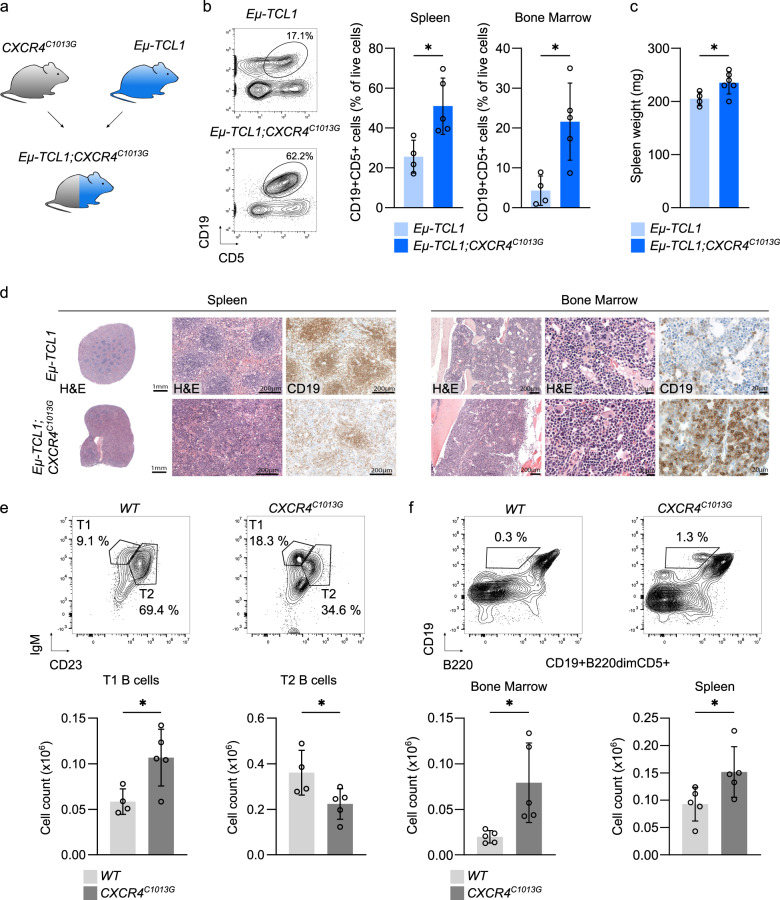
CXCR4 hyperactivation blocks B-cell differentiation leading to expansion of pre-malignant B cells in cooperation with TCL1. **a** Outline of breeding scheme of *CXCR4*^*C1013G*^ and *Eµ-TCL1* mice to generate double-transgenic *Eµ-TCL1;CXCR4*^*C1013G*^ mice. **b** Representative contour plots of splenic CD19+CD5+ cells by flow cytometry in 5–6-month-old animals and quantification of splenic and bone marrow CD19+CD5+ cells (*Eµ-TCL1*, *n* = 4; *Eµ-TCL1;CXCR4*^*C1013G*^, *n* = 5). **c** Spleen weight of 5–6-month-old mice (*Eµ-TCL1*, *n* = 4; *Eµ-TCL1;CXCR4*^*C1013G*^, *n* = 6). **d** Representative images of H&E and immunohistochemistry of 5–6-month-old *Eµ-TCL1* and *Eµ-TCL1;CXCR4*^*C1013G*^ mice (scale bars spleen: overview = 1 mm, detailed images: 200 µm, scale bars bone marrow: overview = 200 µm, detailed images = 20 µm). **e** Quantification and representative contour plots of splenic T1 (IgM+CD23−) and T2 (IgM+CD23+) cells gated on B220+CD93+ B cells by flow cytometry of 3-month-old animals (*WT*, *n* = 5; *CXCR4*^*C1013G*^, *n* = 5). **f** Quantification and representative contour plots of splenic and bone marrow CD19+B220dimCD5+ B1 cells of 3-month-old animals (*WT*, *n* = 5; *CXCR4*^*C1013G*^, *n* = 5). Statistical analyses were performed with Student’s *t* test, *P < 0.05, **P < 0.01, ***P < 0.001. Error bars indicate standard deviation (SD).

CXCR4 plays an essential role in B-cell biology and is highly expressed in B cells as compared to all other cell types (Supplementary Fig. S2a). Considering that CXCR4 hyperactivation induced several cancer-relevant pathways in B cells and confirming the association of elevated CXCR4 expression with adverse prognosis in CLL patients (Supplementary Fig. S2b), we sought to directly investigate the effect of enhanced CXCR4 signaling on B-cell lymphoproliferation and CLL development in vivo. We thus chose the *Eµ-TCL1* model of B-cell tumorigenesis, where the T-cell lymphoma/leukemia 1 (TCL1) transgene is targeted to B cells [21] and intercrossed it with *CXCR4*^*C1013G*^ mice (Fig. 2a). As expected from previous experiments [21], *Eµ-TCL1* mice developed a gradually progressing B-cell lymphoproliferation with a CD19+CD5+/CD19+B220dim immunophenotype (Fig. 2b, Supplementary Fig. S3a). We found that in *Eµ-TCL1;CXCR4*^*C1013G*^ mice, this lymphoproliferation started earlier as compared to *Eµ-TCL1*, indicated by an increased bone marrow and splenic infiltration of CD19+CD5+/CD19+B220dim cells (Fig. 2b, Supplementary Fig. S3a) and increased spleen weight starting at 5–6 months of age (Fig. 2c). This finding was supported by histopathological analysis, where *Eµ-TCl1* mice exhibited enlarged spleens with moderate preservation of the white pulp and clear expansion of the red pulp, which was incipiently infiltrated by B cells, while in *Eµ-TCL1;CXCR4*^*C1013G*^ mice the white pulp was atrophic with expansion of the red pulp and morphologically more pronounced infiltration (Fig. 2d, Supplementary Fig. S3b). In the bone marrow, the infiltration was significantly increased in the *Eµ-TCL1;CXCR4*^*C1013G*^ compared to *Eµ-TCL1* (Fig. 2d, Supplementary Fig. S3b). B220 expression at this stage was significantly lower in *Eµ-TCL1;CXCR4*^*C1013G*^ compared to *Eµ-TCL1* CD19+CD5+ cells (Supplementary Fig. S3c). Importantly, the frequency of CD3e+, CD4+, and CD8+ T cells in spleens of *Eµ-TCL1* and *Eµ-TCL1;CXCR4*^*C1013G*^ did not differ significantly (Supplementary Fig. S3d). In summary, TCL1 mediated B-cell lymphoproliferation is accelerated by enhanced CXCR4 signaling.

To investigate if CXCR4 hyperactivation alone creates a predisposition favoring TCL1-driven B-cell proliferation and CLL development, we performed B-cell immunophenotyping of *CXCR4*^*C1013G*^ mice, focusing on transitional B cells and the population of CD19+/B220dim/CD5+ B1 B cells. A potential mechanism of CLL development in the *Eµ-TCL1* mouse involves accumulation of autoreactive B cells in the transitional T1 population, giving rise to CD19+/B220dim and, to limit autoreactivity, CD5+ B cells, which eventually progress to lethal B-cell leukemia [33–35]. Indeed, in spleens of *CXCR4*^*C1013G*^ mice, T1 B cells were increased, and T2 B cells were decreased (Fig. 2e). The CD19+B220dim/CD5+ B1 B-cell population known from *Eµ-TCL1* mice was already significantly increased in bone marrow and spleen of *CXCR4*^*C1013G*^ mice compared to *WT* littermate controls (Fig. 2f, Supplementary Fig. S3e, f), which was in line with the observed increase in T1 B cells in *Eµ-TCL1;CXCR4*^*C1013G*^. These findings indicate an inherent susceptibility of B cells with CXCR4 hyperactivation for aberrant B-cell lymphoproliferation, which is further enhanced in cooperation with TCL1.

**Fig. 3:**
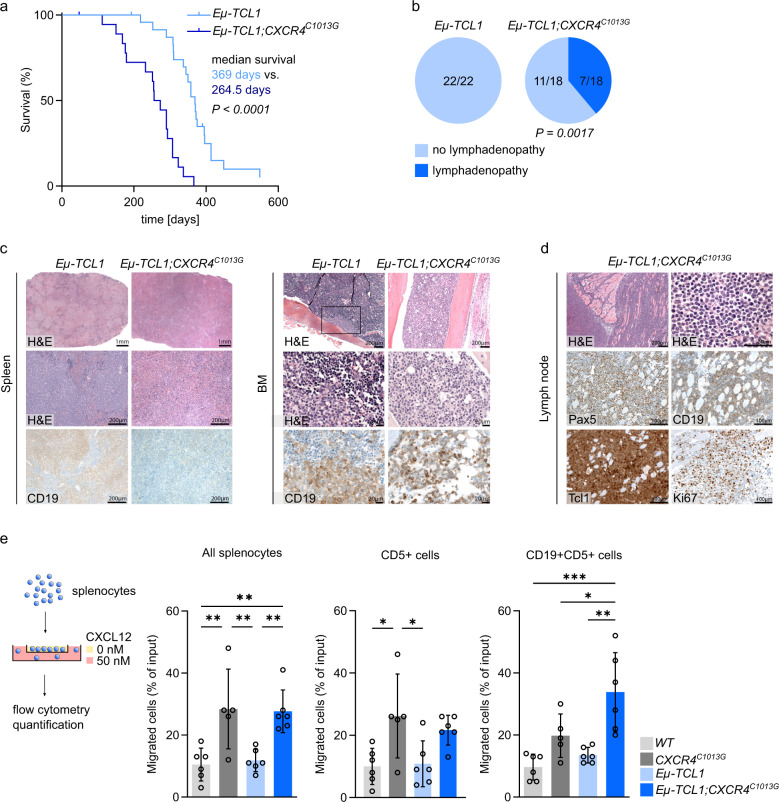
CXCR4 hyperactivation accelerates TCL1-induced lymphomagenesis and dissemination in vivo. **a** Kaplan–Meier survival curves of the indicated cohorts of mice (*Eµ-TCL1*, *n* = 18; *Eµ-TCL1;CXCR4*^*C1013G*^, *n* = 22). Median survival and *P* value of logrank (Mantel–Cox) test is shown. **b** Pie charts depicting fractions of animals with overt lymphadenopathy in *Eµ-TCL1* (*n* = 22) and *Eµ-TCL1;CXCR4*^*C1013G*^ mice (*n* = 18). *P* value of Fisher’s exact test is shown. **c** Representative images of H&E and immunohistochemistry of *Eµ-TCL1* and *Eµ-TCL1;CXCR4*^*C1013G*^ with manifest lymphoma (scale bars spleen: overview = 1 mm, detailed images: 200 µm, scale bars bone marrow: overview = 200 µm, detailed images = 20 µm). **d** Representative images of H&E and immunohistochemistry of a *Eµ-TCL1;CXCR4*^*C1013G*^ animal presenting with Ki67 positive Richter-like aggressive lymphoma (scale bars: H&E overview = 200 µm, H&E detailed image = 50 µm, IHC detailed images = 100 µm). **e** Experimental setup and quantification for ex vivo transwell migration of all splenocytes, CD5+ T cells and CD19+CD5+ cells toward 50 nM CXCL12 isolated from genotypes as indicated (*WT*, *n* = 6; *CXCR4*^*C1013G*^, *n* = 5; *Eµ-TCL1*, *n* = 6; *Eµ-TCL1;CXCR4*^*C1013G*^, *n* = 6). Statistical analyses were performed with one-way ANOVA with Tukey correction for multiple comparisons, *P < 0.05, **P < 0.01, ***P < 0.001. Error bars indicate standard deviation (SD).

Next, we tested in a cohort of *Eµ-TCL1* vs. *Eµ-TCL1;CXCR4*^*C1013G*^ mice how CXCR4 hyperactivation impacts disease onset. We found that CXCR4 activation profoundly accelerates development of symptomatic CLL requiring euthanasia in collaboration with TCL1. Enhancing CXCR4 activity reduced the median survival by ~100 days compared to TCL1 alone (Fig. 3a). Importantly, this finding was accompanied by enhanced nodal dissemination of CLL, as indicated by the development of pronounced lymphadenopathy, which was not observed in *Eµ-TCL1* mice (Fig. 3b, Supplementary Fig. S4a). The immunophenotype and infiltration with T cells remained unchanged in mice with symptomatic CLL requiring euthanasia, as seen in pre-malignant mice, and spleen and liver weights were similar when mice presented with symptoms (Supplementary Figs S4b, c). Histopathology of mice presenting with symptomatic CLL showed extensive infiltration of the red pulp in both genotypes, with complete atrophy of the white pulp in spleens. Visually evaluated by an experienced hematopathologist, the bone marrow of *Eµ-TCL1;CXCR4*^*C1013G*^ mice displayed a diffuse infiltration pattern, compared to a more focal infiltration pattern in *Eµ-TCL1* controls (Fig. 3c, Supplementary Fig. S4d). Strikingly, three out of seven *Eµ-TCL1;CXCR4*^*C1013G*^ mice exhibited hallmark features of aggressive B-cell lymphoma in affected lymph nodes, morphologically resembling DLBCL-type Richter’s transformation occurring in CLL patients. Accordingly, lymphoma cells were larger than typical *Eµ-TCL* CLL cells, infiltrated surrounding muscle and adipose tissue, and showed higher expression of the proliferation marker Ki67 (Fig. 3d and Supplementary Fig. S4e). Furthermore, three *Eµ-TCL1;CXCR4*^*C1013G*^ mice presented with histiocytic sarcoma, a rare complication also in CLL patients, in which CLL cells transform into malignant histiocytes [36] (Supplementary Fig. S4f).

To investigate if the effects of CXCR4 hyperactivation on B-cell leukemia and lymphoma development and dissemination are B cell intrinsic, we isolated splenocytes of pre-malignant animals and assessed their migratory capacity in response to CXCL12 ex vivo. When assessing the subgroup of CD19+CD5+ cells, of which *Eµ-TCL1* tumors evolve, we could see significantly higher migratory capacity specifically in *Eµ-TCL1;CXCR4*^*C1013G*^ compared to *WT*, *Eµ-TCL1* and even *CXCR4*^*C1013G*^. Strikingly, no increased migratory potential compared to *CXCR4*^*C1013G*^ could be seen in bulk splenocytes and CD5+ T cells, suggesting a direct B-cell-intrinsic collaboration of TCL1 and CXCR4 hyperactivation (Fig. 3e).

Thus, we provide first experimental in vivo evidence that CXCR4 hyperactivation supports and accelerates lymphoproliferation and CLL development in a susceptible genetic background, favoring development of a highly proliferative, nodally disseminating cancer with features of aggressive B-cell lymphoma or histiocytic sarcoma in a subset of *Eµ-TCL1;CXCR4*^*C1013G*^ mice.

### Hyperactivated CXCR4 is a hallmark of aggressive lymphoma biology

**Fig. 4:**
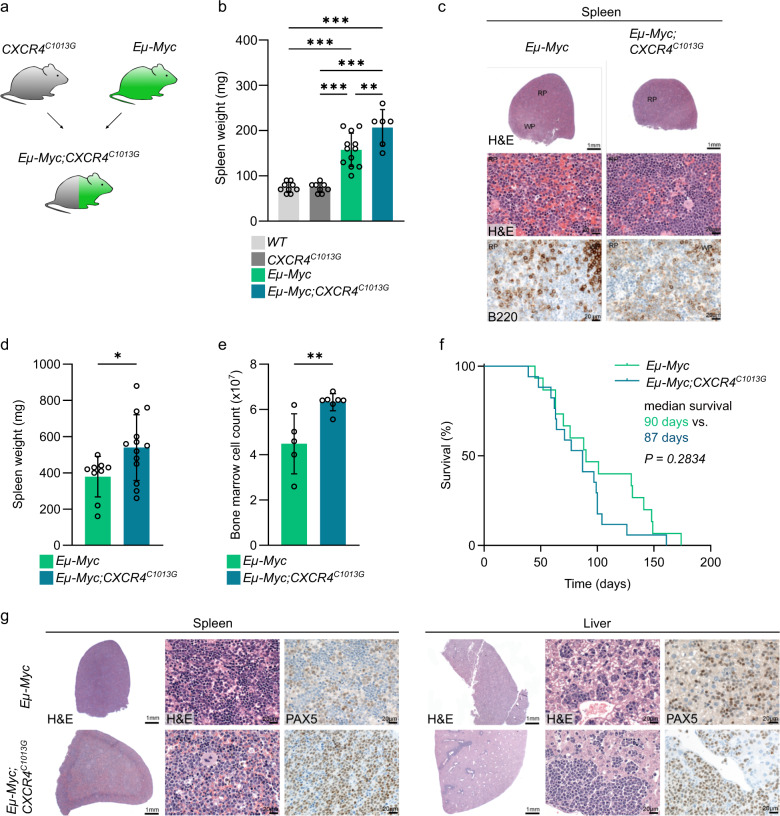
CXCR4 hyperactivation does not accelerate MYC-driven B-cell lymphoma. **a** Outline of breeding scheme of *CXCR4*^*C1013G*^ and *Eµ-Myc* mice to generate double-transgenic *Eµ-Myc;CXCR4*^*C1013G*^ mice. **b** Spleen weight of 1-month-old animals of genotypes as indicated (*WT*, *n* = 9; *CXCR4*^*C1013G*^, *n* = 8; *Eµ-Myc* *n* = 12; *Eµ-Myc;CXCR4*^*C1013G*^, *n* = 6). **c** Representative images of H&E and immunohistochemistry of 1-month-old *Eµ-Myc* and *Eµ-Myc;CXCR4*^*C1013G*^ mice (scale bars: overview = 1 mm, detailed images: 20 µm). **d** Spleen weight of *Eµ-Myc* (*n* = 9) and *Eµ-Myc;CXCR4*^*C1013G*^ (*n* = 13) mice with manifest lymphoma. **e** Bone marrow cell count of *Eµ-Myc* (*n* = 5) and *Eµ-Myc;CXCR4*^*C1013G*^ (*n* = 7) mice with manifest lymphoma. **f** Kaplan–Meier survival curves of the indicated cohorts of mice (*Eµ-Myc*, *n* = 15; *Eµ-Myc;CXCR4*^*C1013G*^, *n* = 17). Median survival and *P* value of logrank (Mantel–Cox) test are shown. **g** Representative images of H&E and immunohistochemistry of *Eµ-Myc* and *Eµ-Myc;CXCR4*^*C1013G*^ mice with manifest lymphoma (scale bars spleen: overview = 1 mm, detailed images: 20 µm, scale bars liver: overview = 1 mm, detailed images = 20 µm). Statistical analyses were performed with Student’s *t* test or one-way ANOVA with Tukey correction for multiple comparisons, *P < 0.05, **P < 0.01, ***P < 0.001. Error bars indicate standard deviation (SD).

Next, we sought to determine if enhanced CXCR4 activity would also result in a more accelerated lymphoma biology in a model of aggressive lymphoma. Analyzing published datasets of DLBCL, the most common aggressive B-cell lymphoma [27, 28, 37], we confirmed that elevated CXCR4 expression correlates with adverse prognosis (Supplementary Fig. S5a) and that *CXCR4* mutations are present, but rare, in DLBCL patients (Supplementary Figure S5b and Supplementary Table T3). To functionally investigate the role of CXCR4 hyperactivation in the context of aggressive B-cell lymphoma, we chose the *Eµ-Myc* mouse model, which is characterized by B-cell-targeted overexpression of MYC, leading to MYC-driven aggressive B-cell lymphoma [31]. This model reflects some of the features of MYC-dependent B-cell biology, which is activated in DLBCL and Burkitt’s lymphoma. To investigate how additional CXCR4 activity alters MYC-induced lymphomagenesis, we intercrossed *CXCR4*^*C1013G*^ with *Eµ-Myc* mice (Fig. 4a). In young 1-month-old mice, we found similarly elevated white blood cell counts in *Eµ-Myc* and *Eµ-Myc;CXCR4*^*C1013G*^ indicating tumor development in both cohorts (Supplementary Fig. S6a). Strikingly, even at this very early time point, we could already discover increased spleen weights compared to age-matched *Eµ-Myc* and control animals (Fig. 4b). This finding was complimented by histopathology, showing that the spleens of *Eµ-Myc;CXCR4*^*C1013*^ mice had a more pronounced infiltration of pre-malignant B cells (Fig. 4c and Supplementary Fig. S6b) very early on. In mice with manifest lymphoma, the *Eµ-Myc;CXCR4*^*C1013G*^ genotype was associated with a more aggressive presentation as demonstrated by larger spleens and higher bone marrow cell count (Fig. 4d, e). Although *Eµ-Myc;CXCR4*^*C1013G*^ displayed features indicative of a more aggressive lymphoma phenotype, median survival did not differ compared to *Eµ-Myc* controls (Fig. 4f). In mice with lymphoma requiring euthanasia, leukocyte counts and immunophenotype of tumors were comparable between both cohorts (Supplementary Fig. S6c, d). Histopathology confirmed the aforementioned findings of the pre-malignant cohorts, and revealed that *Eµ-Myc;CXCR4*^*C1013G*^ mice presented with a higher tendency toward extranodal disease, e.g., hepatic infiltration by lymphoma cells, further indicating a more invasive, extranodal phenotype of CXCR4 hyperactivated lymphoma (Fig. 4g and Supplementary Fig. S6e).

To investigate CXCR4 expression in *Eµ-Myc* mice as compared to *WT* and *Eµ-TCL1* mice as a possible reason for no additional effects of CXCR4 hyperactivation on survival in the *Eµ-Myc* lymphoma model, we first measured CXCR4 surface expression in the different genotypes. *Eµ-Myc* lymphoma cells displayed significantly increased CXCR4 surface expression compared to normal B cells and *Eµ-TCL1* lymphoma cells (Supplementary Fig. S7a), indicating a potential link between MYC and CXCR4 expression. To further explore the association of MYC and CXCR4 expression, we analyzed patient datasets and found a significant correlation of *MYC* mRNA and *IG*-*MYC* translocation status with *CXCR4* mRNA expression in B-cell lymphoma patients (Supplementary Fig. S7b, c). Furthermore, we performed RNA sequencing on CD19+ lymphoma cells from *Eµ-Myc* and *Eµ-Myc;CXCR4*^*C1013G*^ and found that both groups displayed a highly similar transcriptome dominated by the MYC signature, with only eight pathways significantly altered in a gene set enrichment analysis (Supplementary Fig. S8a, b, c). In line with MYC-induced CXCR4 expression, applying our defined signature of CXCR4 hyperactivation did not show significant enrichment in the *Eµ-Myc;CXCR4*^*C1013G*^ versus *Eµ-Myc* (Supplementary Fig. S8d) compared to *Eµ-TCL;CXCR4*^*C1013G*^ versus Eµ-TCL1 (Supplementary Fig. S8e). In *Eµ-TCL;CXCR4*^*C1013G*^ mice the CXCR4 hyperactivation signature was highly and significantly enriched compared to *Eµ-TCL1* littermates. These findings indicate that MYC might positively regulate CXCR4 transcript levels in B-cell lymphoma, which could dampen the additional effect of CXCR4 hyperactivation by *CXCR4*^*C1013G*^.

We conclude that CXCR4 activation is an integrative hallmark of aggressive MYC-driven lymphoma, and show that *MYC* translocation and MYC expression correlate with increased CXCR4 expression. Accordingly, CXCR4^*C1013G*^ in the *Eµ-Myc* model did not further potentiate the already high transcriptional activity mediated by a MYC-CXCR4 axis and had no significant effect on lymphoma latency.

### Co-activation of CXCR4 and TCL1 governs a distinct oncogenic transcriptional program in B cells

**Fig. 5:**
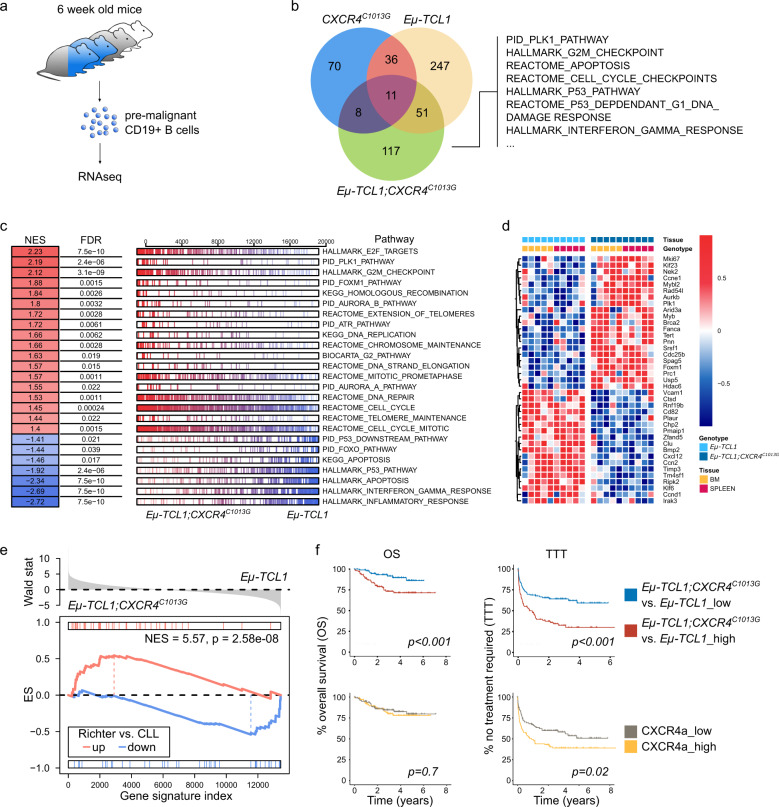
Co-activation of CXCR4 and TCL1 governs a distinct oncogenic transcriptional program in B cells. **a** Outline of experimental setup for RNA sequencing of splenic and bone marrow pre-malignant B cells (*WT*, *n* = 5; *CXCR4*^*C1013G*^, *n* = 5; *Eµ-TCL1*, *n* = 5*; Eµ-TCL1;CXCR4*^*C1013G*^, n = 5). **b** Venn diagram showing overlap of significantly enriched pathways (padj < 0.05) for curated gene sets listed in MsigDB [63, 64] of indicated genotypes vs. *WT* B cells as determined by gene set enrichment analysis (GSEA). **c** GSEA of *Eµ-TCL1;CXCR4*^*C1013G*^ vs. *Eµ-TCL1* CD19+ B cells showing normalized enrichment scores (NES) and false discovery rates (FDR) for curated gene sets listed in MsigDB [63, 64]. **d** Selection of differentially expressed genes (DEG) with adjusted *P* value (padj) < 0.05 of *Eµ-TCL1;CXCR4*^*C1013G*^ vs. *Eµ-TCL1* CD19+ B cells. **e** GSEA showing enrichment of a Richter’s transformation signature [43] in *Eµ-TCL1;CXCR4*^*C1013G*^ B cells. **f** Kaplan–Meier plots of overall survival (OS) and time to treatment (TTT) in a cohort of CLL patients, for patients with high vs. low enrichment of upregulated genes from CXCR4 signatures (*CXCR4*^*C1013G*^ vs. *WT* and *Eµ-TCL1;CXCR4*^*C1013G*^ vs. *Eµ-TCL1*) (TTT *CXCR4*^*C1013G*^ vs. *WT*, *n* = 105 high, *n* = 103 low; OS *CXCR4*^*C1013G*^ vs. *WT* and *Eµ-TCL1;CXCR4*^*C1013G*^ vs. *Eµ-TCL1*, n = 105 in each group; TTT *Eµ-TCL1;CXCR4*^*C1013G*^ vs. *Eµ-TCL1*, *n* = 104 in each group). *P* values of logrank (Mantel–Cox) test are shown.

To further investigate how enhanced CXCR4 activation promotes B-cell lymphoproliferation in vivo, we isolated CD19+ B cells from spleen and bone marrow of 6-week-old *Eµ-TCL1* and *Eµ-TCL1;CXCR4*^*C1013G*^ mice and respective controls and performed whole-transcriptome profiling (Fig. 5a). As expected, neither *Eµ-TCL1* nor *Eµ-TCL1;CXCR4*^*C1013G*^ had monoclonal B-cell proliferation at this stage detectable by transcriptomic clonality analysis (Supplementary Fig. S9) [38–40]. Gene set enrichment analysis of curated pathways in *CXCR4*^*C1013G*^*, Eµ-TCL1*, and *Eµ-TCL1;CXCR4*^*C1013G*^ compared to *WT* controls revealed an overlap of deregulated pathways (Fig. 5b). Importantly, pathways associated with B-cell cancer biology and inflammation were enriched and DNA repair pathways were depleted in *Eµ-TCL1* compared to *WT* B cells, confirming the pre-malignant state of *Eµ-TCL1* B cells (Supplementary Fig. S10a). A subset of deregulated pathways were enriched specifically in *Eµ-TCL1;CXCR4*^*C1013G*^ B cells (Fig. 5b). Among those, pathways involved in cell cycle progression (PLK1 pathway, G2M checkpoint, cell cycle checkpoints) were enriched, whereas pathways including p53 signaling and apoptosis (P53 pathway, P53 dependent G1 DNA damage response, apoptosis) and immune response (interferon gamma response) were depleted in *Eµ-TCL1;CXCR4*^*C1013G*^ B cells.

Next, to gain further insight into the phenotypic differences and disease acceleration caused by additional CXCR4 signaling, we directly compared *Eµ-TCL1* with *Eµ-TCL1;CXCR4*^*C1013G*^ B cells. Strikingly, we found that the transcriptome of *Eµ-TCL1;CXCR4*^*C1013G*^ B cells maintained a distinct profile even compared to *Eµ-TCL1*, comprising enrichment of cell cycle progression and proliferation pathways and depletion of p53-, apoptosis-, and immune response-related pathways (Fig. 5c). Analyzing expression of individual genes, we found 2140 differentially expressed genes between pre-malignant B cells from *Eµ-TCL1* vs. *Eµ-TCL1;CXCR4*^*C1013G*^ mice. In line with the gene set enrichment analysis, cell cycle genes like cyclins (Ccne1), cyclin-dependent kinase phosphatases (*Cdc25b*), mitotic regulators (*Aurkb, Prc1, Hdac6, Nek2, Mki67*), DNA repair and DNA damage response genes (*Brca2, Fanca, Rad54l*) were among the top differentially expressed genes. Transcripts of well-established tumor suppressor genes (*Klf6, CD82*) and positive regulators of apoptosis (*Pmaip1*) were found to be suppressed in *Eµ-TCL1;CXCR4*^*C1013G*^ (Fig. 5d). Remarkably, multiple key components of the Plk1-Foxm1 pathway were among the top upregulated genes, including *Plk1*, *Foxm1* itself, the bona-fide *Foxm1* target *Cdc25b*, the Foxm1 deubiquitinating enzyme *Usp5* involved in stabilization of Foxm1 protein [41], and *Myb*, which forms a complex with Foxm1 and is required for its function.

To further support the finding that CXCR4 hyperactivation potentiates oncogenic programs, we used the Cancer Gene Census [42] and human B-cell lymphoma datasets [27, 28] for cross-species validation. We calculated the overlap of DEG in *Eµ-TCL1;CXCR4*^*C1013G*^ vs. *Eµ-TCL1* B cells with the Cancer Gene Census (Supplementary Fig. S10b) and DLBCL driving genes in two large patient cohorts (Supplementary Fig. S10c) and found that ten DEGs in our cohort were identified in both DLBCL datasets [27, 28].

To further evaluate if the transcriptional program of patients with Richter’s transformation resembles the transcriptomic changes supported by enhanced CXCR4 signaling, we generated a gene signature from CLL patient data with Richter’s transformation [43]. Strikingly, this “Richter-signature” enriched significantly only in *Eµ-TCL1;CXCR4*^*C1013G*^, but not in *Eµ-TCL1* or *CXCR4*^*C1013G*^ B cells, showing that key transcriptomic features of Richter’s transformation are promoted by CXCR4 hyperactivation in cooperation with TCL1 (Fig. 5e and Supplementary Fig. S10d). Finally, we stratified a cohort of CLL patients using upregulated genes of the *CXCR4*^*C1013G*^ vs. *WT* (CXCR4a) and *Eµ-TCL1;CXCR4*^*C1013G*^ vs *Eµ-TCL1* signature. We found that patients characterized by CXCR4a had significantly reduced time to treatment (TTT) and patients characterized by the *Eµ-TCL1;CXCR4*^*C1013G*^ vs *Eµ-TCL1* signature had significantly reduced TTT and overall survival (Fig. 5f), further supporting the association of CXCR4 hyperactivation with aggressive lymphoma biology.

## Discussion

In this study we co-expressed *CXCR4*^*C1013G*^ with the B-cell oncogenes *TCL1* and *MYC* to investigate the effects of enhanced CXCR4 signaling on B-cell leukemogenesis and lymphomagenesis. Hyperactivated CXCR4 functioned as an oncogene cooperating with TCL1 to accelerate CLL progression and development of aggressive B-cell lymphoma.

CXCR4 expression has been implicated as an adverse prognostic factor in DLBCL in retrospective analyses [14, 44]. However, CXCR4 signaling activity is a highly dynamic and tightly regulated process, which is not always adequately reflected by gene expression alone [5]. Previous studies on CXCR4 gain-of-function mutations focused on established Waldenström Macroglobulinemia cell lines, as CXCR4 mutations are present in a third of patients with this disease [45], thereby limiting conclusions to already transformed cells [46, 47]. The role of CXCR4 in CLL has been thoroughly investigated and it has become clear that the CXCR4-CXCL12 axis is essential for CLL cells and their interaction with the microenvironment, especially in response to BTK inhibition with ibrutinib [17, 25]. However, it was unclear how hyperactive CXCR4 signaling impacts the development of CLL in vivo. Our work represents the first study providing in vivo evidence that *CXCR4* acts as an oncogene in B cells by expressing a gain-of-function mutation together with known B-cell oncogenes. Importantly, CXCR4^*C1013G*^ provokes tremendous transcriptomic and phenotypic changes in pre-malignant B cells. We discovered an expansion of B1 B cells mediated by CXCR4^*C1013G*^, indicating a susceptibility of hyperactivated CXCR4 signaling toward mature B-cell neoplasms, as this fraction of B cells harbor the potential for transformation into CLL and mantle cell lymphoma [48]. This susceptibility does not result in development of B-cell neoplasms in the *CXCR4*^*C1013G*^ mouse model alone [9] but requires the context of additional oncogenic drivers, as shown in our studies. In cooperation with the oncogene TCL1, CXCR4 hyperactivation even favors development of aggressive B-cell lymphoma. Intriguingly, in CLL patients with Richter’s transformation mutations in regulatory elements of *CXCR4* have recently been discovered [43].

The transcriptional alterations induced by CXCR4 hyperactivation were consistent with CXCR4 function as indicated by enrichment of curated pathway gene sets for chemokine signaling and inflammation. Intriguingly, RNA sequencing also revealed enhanced expression of *Prdm1*, a master regulator of plasma cell proliferation and differentiation, which was found to be essential for the survival of WM cells by regulating EZH2, a promising therapeutic target in B-cell lymphoma [49, 50].

Of note, no mouse model with B-cell-specific *CXCR4*^*C1013G*^ expression exists, therefore additional, non-B-cell-intrinsic effects of transgene expression in the microenvironment need to be taken into consideration. It cannot be completely ruled out that acceleration of lymphoma development might be partially mediated by activated CXCR4 signaling in T cells or other cell types of the tumor microenvironment known to express CXCR4. Importantly, all transcriptomic data shown originate from CD19-purified B cells. The finding that *CXCR4* gene expression is highest in B cells, and data that identified CXCR4 as a major regulator of B-cell development and function [2, 10, 51, 52] further support the notion of a B-cell-intrinsic effect of CXCR4 signaling in our models. Furthermore, the migratory capacity ex vivo, without any support of the microenvironment, is enhanced most prominently in CD19+CD5+ B cells of double-transgenic *Eµ-TCL1;CXCR4*^*C1013G*^ animals compared to controls, suggesting a direct collaboration of TCL1 and CXCR4 in a specific subset of B cells. Of note, frequency of CD4+ and CD8+ T cells in spleens of *Eµ-TCL1* and *Eµ-TCL1;CXCR4*^*C1013G*^ did not differ significantly, which might suggest that CXCR4 activation in T cells did not result in dramatically reduced T-cell-mediated immune surveillance. Moreover, we could detect an aggressive lymphoma phenotype in the histology of a subset of mice as well as an enrichment of a patient-derived “Richter-signature” in the transcriptomic program of pre-malignant *Eµ-TCL1;CXCR4*^*C1013G*^ B cells. This further indicates a B-cell-specific influence of CXCR4 signaling toward a more aggressive lymphoma phenotype. In the light of recent findings linking active Akt signaling, a downstream target of CXCR4, to Richter’s transformation [23], it seems possible that Akt phosphorylation by hyperactivated CXCR4 is a contributing factor for the aggressive lymphoma phenotype in our CXCR4 hyperactivation models.

Using the *Eµ-Myc* lymphoma model, we showed that CXCR4 transcript and surface expression is enhanced by oncogenic MYC. MYC can bind and activate the CXCR4 promoter [53], but this connection has not been explored in B-cell lymphoma so far. Young *Eµ-Myc;CXCR4*^*C1013G*^ animals presented with increased spleen weight and more pronounced lymphoproliferation, which did not translate in a reduction in overall survival. This could be explained by a proportion of transformed B cells depending fully on the MYC-oncogenic program and exponential tumor growth in *Eµ-Myc* animals, which could mask biologically relevant effects of additional CXCR4 hyperactivation as used in our experimental approach. Furthermore, data from large B-cell lymphoma patient datasets revealed a correlation of MYC and CXCR4 expression and an increase of CXCR4 expression in patient samples with translocation of *MYC* further supporting a regulation of CXCR4 by MYC in a B-cell oncogenic context.

The cooperation of CXCR4 and TCL1 was associated with transcriptional activation of the Foxm1-Plk1 axis and proliferative pathways [54]. PI3K and Akt might be the central signaling node connecting CXCR4 and TCL1 signaling, resulting in inhibition of Foxo3 and de-repression of Foxm1 [55, 56]. In addition, PI3K/Akt phosphorylates Plk1 that engages in a feed-forward loop with Foxm1 [57]. Both Plk1 and Foxm1 are promising therapeutic targets in B-cell lymphoma [58, 59]. The Myb oncogene, which also displays increased transcript expression in our *Eµ-TCL1;CXCR4*^*C1013G*^ vs. *Eµ-TCL1* RNA-Seq data, is crucial for Foxm1 function [60, 61], and the Myb-Foxm1 interaction is required for proliferation in the germinal centers, a site prone for malignant transformation of B cells [62].

In summary, we identified hyperactivated CXCR4 signaling as a cooperative oncogenic factor in B-cell leukemogenesis and lymphomagenesis associated with a distinct transcriptional program. Hyperactivated CXCR4 resulted in a more disseminated lymphoma phenotype and accelerated disease in TCL1-driven CLL, while also favoring development of aggressive lymphoma. Next to its role as a prognostic factor, CXCR4 activation might serve as potential biomarker for novel targeted therapies, e.g., therapies targeting the FOXM1-PLK1 axis and EZH2. These findings warrant further investigation.

## Supplementary information

Supplemental Information

Supplemental Figure S1

Supplemental Figure S2

Supplemental Figure S3

Supplemental Figure S4

Supplemental Figure S5

Supplemental Figure S6

Supplemental Figure S7

Supplemental Figure S8

Supplemental Figure S9

Supplemental Figure S10

Supplemental Table T1

Supplemental Table T2

Supplemental Table T3

## Data Availability

The RNA-Seq data reported in this study are available on the National Center for Biotechnology Information’s Gene Expression Omnibus under the accession code GSE178959.
