# Supplementary material for: CXCR4 hyperactivation cooperates with TCL1 in CLL development and aggressiveness
Source: Leukemia. 2021 Aug 6;35(10):2895–905. doi: 10.1038/s41375-021-01376-1 (PMC8478649; doi:10.1038/s41375-021-01376-1)
Supplement: Supplementary file 1 — Supplemental Information [file 41375_2021_1376_MOESM1_ESM.docx]

**SUPPLEMENTAL DATA**

Supplemental data include supplemental methods, supplemental references and supplemental figures S1-S10.

**SUPPLEMENTAL METHODS**

### **Scoring of immunohistochemistry**

To quantify immunohistochemistry, we established a scoring system accessing the tumor infiltration for each organ (see below). All samples were then scored by an experienced hematopathologist (Leticia Quintanilla-Martinez), accordingly.

Spleen:

Score 1: Preserved white pulp and beginning of infiltration in the red pulp.

Score 2: Decreased white pulp and increase in red pulp infiltration.

Score 3: Loss of white pulp and complete red pulp infiltration.

Liver:

Score 1: Isolated positive cells.

Score 2: Intrasinusoidal infiltration or few portal small groups of lymphoid cells.

Score 3: Intrasinusoidal and portal infiltration with large groups of lymphoid cells.

Bone marrow:

Score 1: Isolated positive cells.

Score 2: Interstitial infiltration or few small groups of lymphoid cells.

Score 3: Interstitial infiltration and large groups of lymphoid cells.

### **RNA sequencing**

*Eµ-Myc* and *Eµ-Myc;CXCR4^C1013G^* samples: Briefly RNA sequencing of *Eµ-Myc*, *Eµ-Myc;CXCR4^C1013G^* was performed on a NextSeq 500 (Illumina, San Diego, CA) as described by Parekh, Ziegenhein et al. and using the adjusted protocol as described previously by Scherger, Al-Maarri et al. (1, 2).

### **Differential gene expression analysis**

Genome-wide differential gene expression analysis for RNA-Seq count data was carried out using a negative binomial generalized linear model (GLM) as implemented in the DESeq2 R package (3)(4) to test for differentially expressed genes between experimental conditions. For dispersion estimation we considered genotype and organ site as covariates. For individual comparisons a false discovery rate (FDR) < 0.1 was considered significant. Select differentially

expressed genes were illustrated in a heatmap after rescaling to have a minimum of -1 and a maximum of +1 using the *pheatmap*  R package (5).

### **Gene Set Enrichment Analysis**

Gene set enrichment analysis (GSEA) was carried out on individual differential gene expression signatures between two conditions using the *fgsea* (6) R package and using Wald statistics from the aforementioned GLM as gene-level statistics. Gene sets were retrieved from the MSigDb v7.3 (7, 8) Enrichment results for select pathways were illustrated using custom R code.

### **Derivation and application of CXCR4 associated gene signatures**

First we carried out differential gene expression (DEG) analysis on *WT, CXCR4^C1013G^*, *Eµ-TCL1* and *Eµ-TCL1;CXCR4^C1013G^* B cells as described. Next, we applied the following thresholds to the DEG results: baseMean > 50, absolute log_2_ fold change > 0.5 and adjusted p-value < 0.05. The remaining genes were ordered by their absolute Wald test statistic for up- (i.e. induced by CXCR4 activation) and downregulated (i.e. repressed by CXCR4 activation) genes, respectively, to represent the CXCR4 activation signature (Supplemental Table T2). Its enrichment on gene expression signatures derived from comparing other experimental conditions was computed using analytic rank-based enrichment analysis (aREA) (9) as implemented in the viper (10) R package considering both tails of the CXCR4a activation signature. Similarly, we applied differentially expressed genes between Richter transformed lymphocytes and peripheral blood chronic lymphocytic leukemia (11) to gene expression signatures derived from our experimental conditions.

**Application of upregulated genes from CXCR4 associated gene signatures to a data set of CLL patients**

Simple sample gene set enrichment analysis (ssGSEA) using GSVA R/Bioconductor package (12) was performed on a previously published RNA sequencing dataset derived from a cohort of 210 CLL patients (13). For each patient sample, an enrichment score was calculated based on the upregulated genes from our CXCR4a (*CXCR4^C1013G^* vs. *WT*) and *Eµ-TCL1;CXCR4^C1013G^* vs. *Eµ-TCL1* signature and patients were dichotomized into two equally sized groups of high and low enrichment along the median. Groups were compared regarding their overall survival and time to treatment using log-rank test and the difference was visualized by Kaplan-Meier plots.

**Overlapping DEGs with known datasets of oncogenes**

DEGs, as described above, of 6 week old *Eµ-TCL1* and *Eµ-TCL1;CXCR4^C1013G^* were generated and pre-selected for protein coding genes with exclusion of Riken-cDNA genes using the MGI database. (14). We then overlapped the DEGs with the Cancer Gene Census dataset (15) of known cancer oncogenes and two data sets of published B cell oncogenes (16, 17).

**Clonality Analysis from transcriptomic data**

Transcriptomic data generated from RNA-Sequencing of CD19+ B cells from 6 week old mice was used to profile V(D)J rearrangement using MiXCR and VDJtools as previously published (18, 19). Briefly, adapters were trimmed using Trim Galore (RRID:SCR_011847). Subsequently the paired-end set of reads were loaded into the MiXCR analysis pipeline (shotgun analysis) using a built-in mouse library (19). Finally, clonotype results from MiXCR were analyzed and visualized using VDJtools.

**SUPPLEMENTAL REFERENCES**

1. Scherger AK, Al-Maarri M, Maurer HC, Schick M, Maurer S, Ollinger R, et al. Activated gp130 signaling selectively targets B cell differentiation to induce mature lymphoma and plasmacytoma. JCI Insight. 2019;4(15).

2. Parekh S, Ziegenhain C, Vieth B, Enard W, Hellmann I. The impact of amplification on differential expression analyses by RNA-seq. Sci Rep. 2016;6:25533.

3. R Core Team. R: A Language and Environment for Statistical Computing. <https://www.R-project.org/> (2018).

4. Love MI, Huber W, Anders S. Moderated estimation of fold change and dispersion for RNA-seq data with DESeq2. Genome Biol. 2014;15(12):550.

5. Kolde, R. pheatmap: Pretty Heatmaps. <https://CRAN.R-project.org/package=pheatmap> (2019).

6. Korotkevich, G. et al. Fast gene set enrichment analysis. bioRxiv (2021) doi:10.1101/060012

7. Liberzon A, Subramanian A, Pinchback R, Thorvaldsdottir H, Tamayo P, Mesirov JP. Molecular signatures database (MSigDB) 3.0. Bioinformatics. 2011;27(12):1739-40.

8. Subramanian A, Tamayo P, Mootha VK, Mukherjee S, Ebert BL, Gillette MA, et al. Gene set enrichment analysis: a knowledge-based approach for interpreting genome-wide expression profiles. Proc Natl Acad Sci U S A. 2005;102(43):15545-50.

9. Alvarez MJ, Shen Y, Giorgi FM, Lachmann A, Ding BB, Ye BH, et al. Functional characterization of somatic mutations in cancer using network-based inference of protein activity. Nat Genet. 2016;48(8):838-47.

10. Alvarez, M. J., Giorgi, F. & Califano, A. Using viper, a package for Virtual Inference of Protein-activity by Enriched Regulon analysis. Bioconductor 1–14 (2014).

11. Klintman J, Appleby N, Stamatopoulos B, Ridout K, Eyre TA, Robbe P, et al. Genomic and transcriptomic correlates of Richter's transformation in Chronic Lymphocytic Leukemia. Blood. 2020.

12. Hänzelmann S, Castelo R, Guinney J. GSVA: gene set variation analysis for microarray and RNA-Seq data. BMC Bioinformatics. 2013;14(1):7.

13. Dietrich S, Oleś M, Lu J, Sellner L, Anders S, Velten B, et al. Drug-perturbation-based stratification of blood cancer. The Journal of Clinical Investigation. 2018;128(1):427-45.

14. Bult CJ, Blake JA, Smith CL, Kadin JA, Richardson JE, Mouse Genome Database G. Mouse Genome Database (MGD) 2019. Nucleic Acids Res. 2019;47(D1):D801-D6.

15. Sondka Z, Bamford S, Cole CG, Ward SA, Dunham I, Forbes SA. The COSMIC Cancer Gene Census: describing genetic dysfunction across all human cancers. Nat Rev Cancer. 2018;18(11):696-705.

16. Reddy A, Zhang J, Davis NS, Moffitt AB, Love CL, Waldrop A, et al. Genetic and Functional Drivers of Diffuse Large B Cell Lymphoma. Cell. 2017;171(2):481-94 e15.

17. Chapuy B, Stewart C, Dunford AJ, Kim J, Kamburov A, Redd RA, et al. Molecular subtypes of diffuse large B cell lymphoma are associated with distinct pathogenic mechanisms and outcomes. Nat Med. 2018;24(5):679-90.

18. Shugay M, Bagaev DV, Turchaninova MA, Bolotin DA, Britanova OV, Putintseva EV, et al. VDJtools: Unifying Post-analysis of T Cell Receptor Repertoires. PLoS Comput Biol. 2015;11(11):e1004503.

19. Bolotin DA, Poslavsky S, Mitrophanov I, Shugay M, Mamedov IZ, Putintseva EV, et al. MiXCR: software for comprehensive adaptive immunity profiling. Nat Methods. 2015;12(5):380-1.
