# Supplementary material for: CXCR4 hyperactivation cooperates with TCL1 in CLL development and aggressiveness
Source: Leukemia. 2021 Aug 6;35(10):2895–905. doi: 10.1038/s41375-021-01376-1 (PMC8478649; doi:10.1038/s41375-021-01376-1)
Supplement: Supplementary file 2 — Supplemental Figure S1 [file 41375_2021_1376_MOESM2_ESM.pdf]

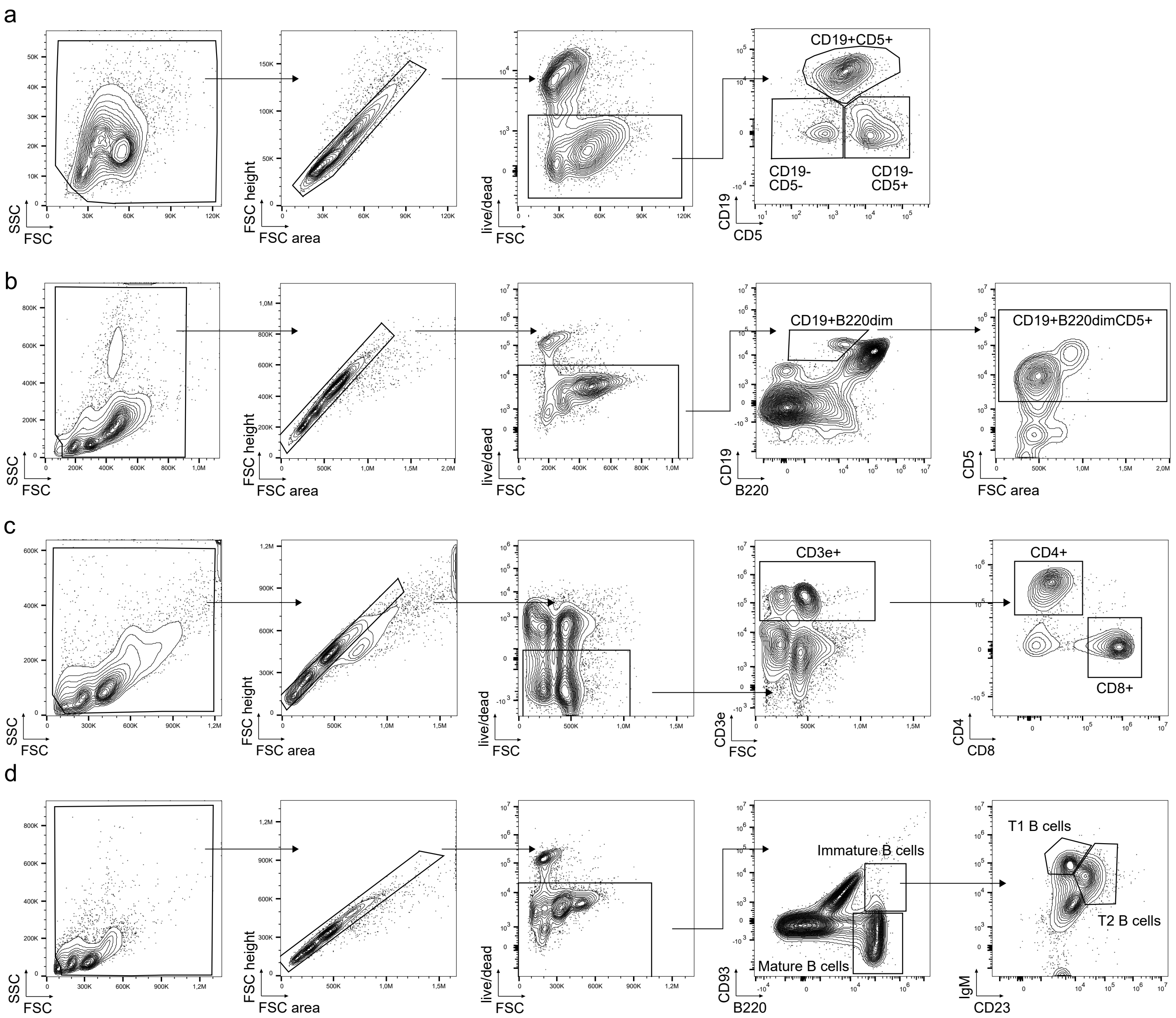

Supplemental Figure S1. Flow cytometry gating strategies.

(a) Gating strategy for CD19+CD5+ cells.

(b) Gating strategy for CD19+B220dim cells and CD19+B220dimCD5+ B1 B cells.

(c) T cell gating strategy to measure frequency of all T cells (CD3e+) and to distinguish between CD4+ and CD8+ cells.

(d) Gating strategy for transitional T1 (IgM+CD23-) and T2 (IgM+CD23+) cells gated on B220+CD93+.
