# Supplementary material for: CXCR4 hyperactivation cooperates with TCL1 in CLL development and aggressiveness
Source: Leukemia. 2021 Aug 6;35(10):2895–905. doi: 10.1038/s41375-021-01376-1 (PMC8478649; doi:10.1038/s41375-021-01376-1)
Supplement: Supplementary file 3 — Supplemental Figure S2 [file 41375_2021_1376_MOESM3_ESM.pdf]

a

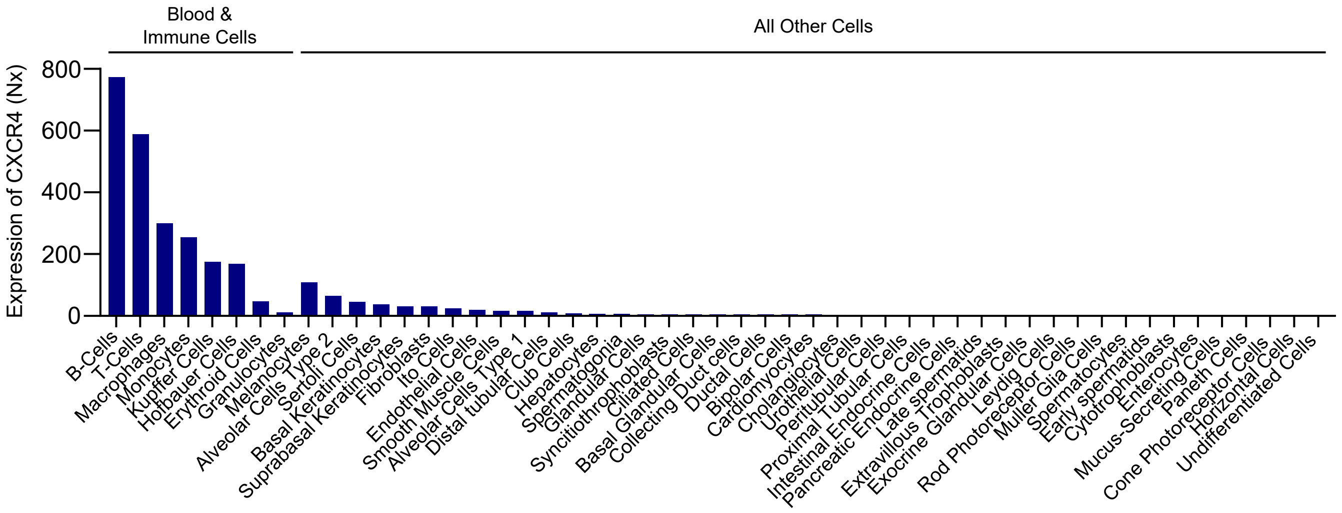

b

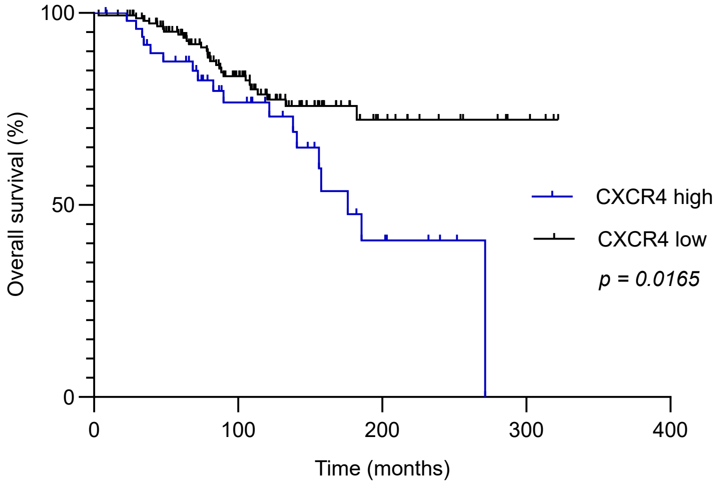

Supplemental Figure S2. High CXCR4 expression is associated with adverse prognosis in CLL patients.
(a) Comparison of physiologic expression levels of CXCR4 mRNA among different cell types obtained from the Human Protein Atlas, shown as Nx expression scores of single cell transcriptomic data (20).
(b) Kaplan-Meier survival analysis of 201 CLL patients stratified by CXCR4 mRNA expression from the CLL-ES data set of the ICGC database – June 2016, risk-group maximized and analyzed with SurvExpress (21). P value of log-rank (Mantel-Cox) test is shown.
