# Supplementary material for: CXCR4 hyperactivation cooperates with TCL1 in CLL development and aggressiveness
Source: Leukemia. 2021 Aug 6;35(10):2895–905. doi: 10.1038/s41375-021-01376-1 (PMC8478649; doi:10.1038/s41375-021-01376-1)
Supplement: Supplementary file 4 — Supplemental Figure S3 [file 41375_2021_1376_MOESM4_ESM.pdf]

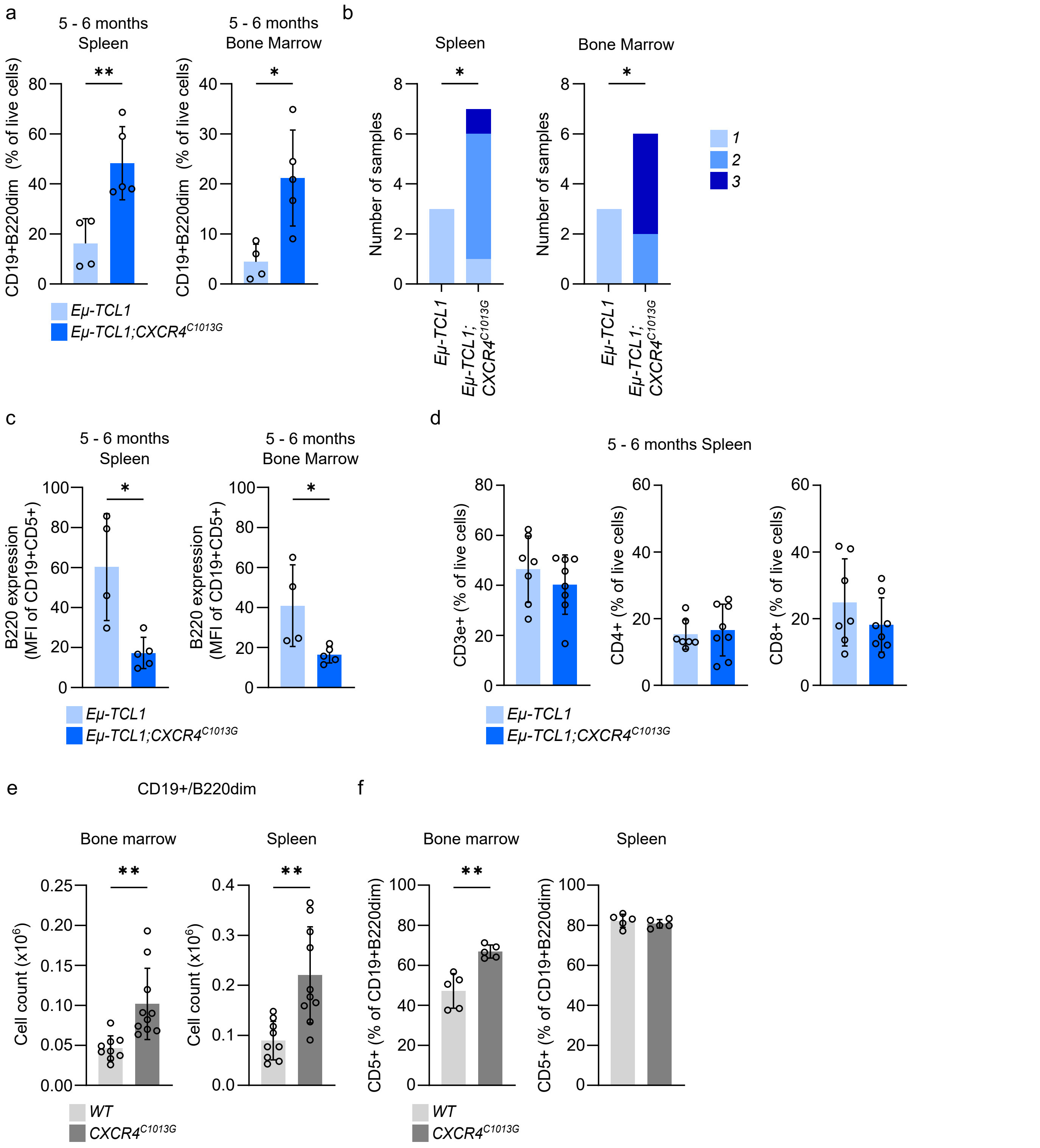

Supplemental Figure S3. Characterization of the *Eμ-TCL1;CXCR4*^{C1013G} and *CXCR4*^{C1013G} phenotype.

(a) Frequencies of spleen and bone marrow CD19+B220dim cells by flow cytometry in 5-6 month old animals (*Eμ-TCL1*, n = 4; *Eμ-TCL1;CXCR4*^{C1013G}, n = 5).

(b) Quantification of histology scores (1-3) of spleen (*Eμ-TCL1*, n = 3; *Eμ-TCL1;CXCR4*^{C1013G}, n = 7) and bone marrow (*Eμ-TCL1*, n = 3; *Eμ-TCL1;CXCR4*^{C1013G}, n = 6) of 5 - 6 month old mice. Scoring system details are shown in Supplemental methods.

(c) Quantification of spleen and bone marrow B220 expression of CD19+CD5+ cells by flow cytometry in 6 month old animals (*Eμ-TCL1*, n = 4; *Eμ-TCL1;CXCR4*^{C1013G}, n = 5).

(d) Frequencies of spleen CD3e+, CD4+ and CD8+ T cells by flow cytometry in 5-6 month old animals (*Eμ-TCL1*, n = 7; *Eμ-TCL1;CXCR4*^{C1013G}, n = 8).

(e) Quantification of total CD19+B220dim cells in bone marrow and spleen of 3 month old *WT* and *CXCR4*^{C1013G} mice (*WT*, n = 5; *CXCR4*^{C1013G}, n = 5).

(f) Frequencies of CD5+ cells of CD19+B220dim cells in bone marrow and spleen of 3 month old *WT* and *CXCR4*^{C1013G} mice (*WT*, n = 5; *CXCR4*^{C1013G}, n = 5).

Statistical analyses were performed with Student's t test (a, b, c, e, f) or Mann-Whitney *U* test (d), * $P < 0.05$, ** $P < 0.01$. Error bars indicate standard deviation (SD).
