# Supplementary material for: CXCR4 hyperactivation cooperates with TCL1 in CLL development and aggressiveness
Source: Leukemia. 2021 Aug 6;35(10):2895–905. doi: 10.1038/s41375-021-01376-1 (PMC8478649; doi:10.1038/s41375-021-01376-1)
Supplement: Supplementary file 5 — Supplemental Figure S4 [file 41375_2021_1376_MOESM5_ESM.pdf]

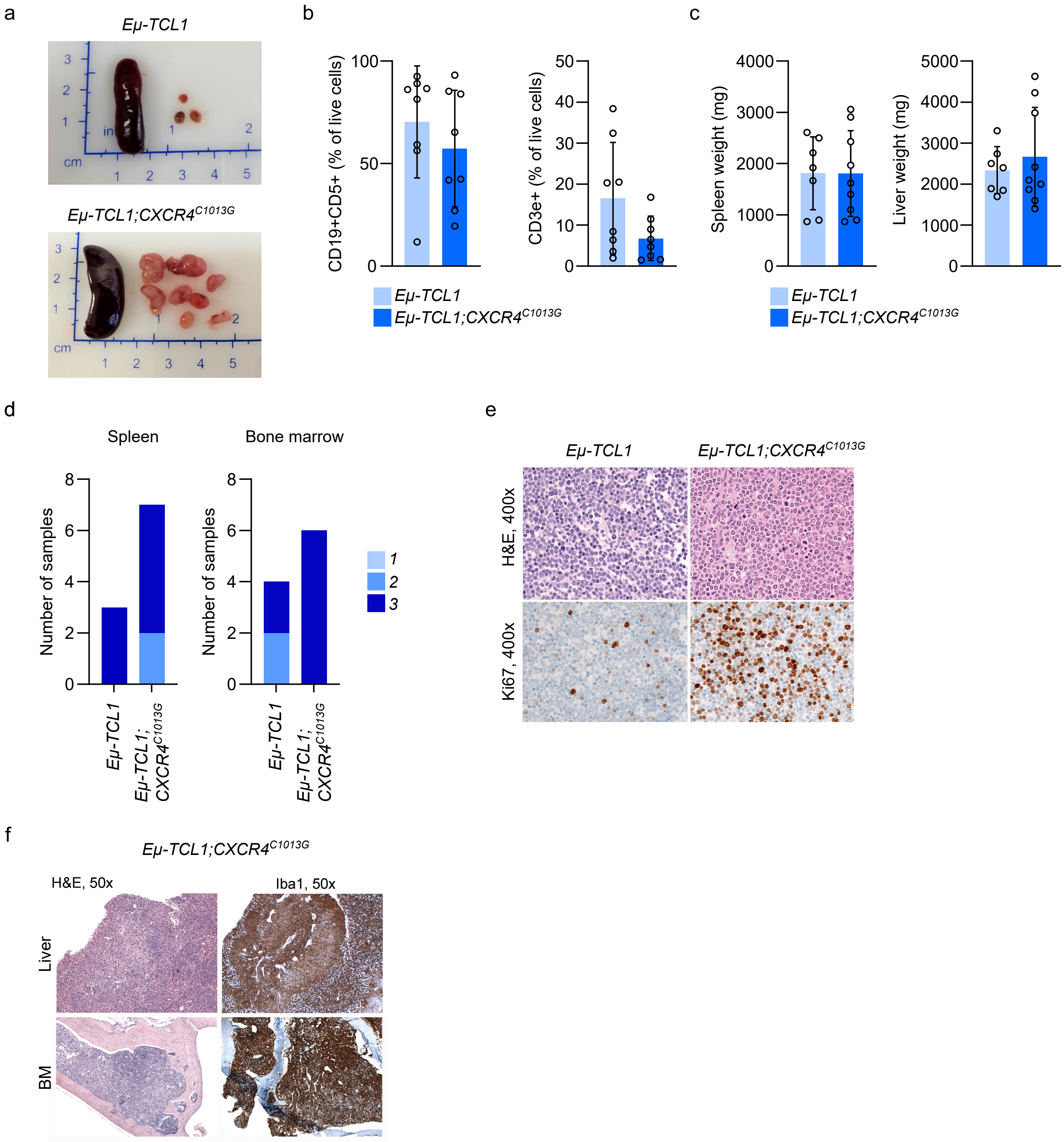

Supplemental Figure S4. Characterization of *Eμ-TCL1;CXCR4*^{C1013G} with symptomatic CLL requiring euthanasia.

(a) Representative images of spleen and lymphatic tissue of mice with symptomatic CLL requiring euthanasia of a *Eμ-TCL1* control and a *Eμ-TCL1;CXCR4*^{C1013G} animal presenting with extensive lymphadenopathy.

(b) Frequencies of spleen CD19+CD5+ and CD3e+ cells by flow cytometry in mice with symptomatic CLL requiring euthanasia (*Eμ-TCL1*, n = 8; *Eμ-TCL1;CXCR4*^{C1013G}, n = 8).

(c) Spleen and liver weight of mice with symptomatic CLL requiring euthanasia (*Eμ-TCL1*, n = 7; *Eμ-TCL1;CXCR4*^{C1013G}, n = 9).

(d) Quantification of histology scores (1-3) of spleen (*Eμ-TCL1*, n = 3; *Eμ-TCL1;CXCR4*^{C1013G}, n = 7) and bone marrow (*Eμ-TCL1*, n = 4; *Eμ-TCL1;CXCR4*^{C1013G}, n = 6) of mice with symptomatic CLL requiring euthanasia. Scoring system details are shown in Supplemental Methods.

(e) Representative images of aggressive lymphoma morphology in *Eμ-TCL1;CXCR4*^{C1013G} animals compared to *Eμ-TCL1* animals with symptomatic CLL requiring euthanasia.

(f) Representative images of histiocytic sarcoma in *Eμ-TCL1;CXCR4*^{C1013G} animals.

Statistical analyses were performed with Student's t test (b and c) or Mann-Whitney U test (d), * $P < 0.05$, ** $P < 0.01$. Error bars indicate standard deviation (SD).
