# Supplementary material for: CXCR4 hyperactivation cooperates with TCL1 in CLL development and aggressiveness
Source: Leukemia. 2021 Aug 6;35(10):2895–905. doi: 10.1038/s41375-021-01376-1 (PMC8478649; doi:10.1038/s41375-021-01376-1)
Supplement: Supplementary file 6 — Supplemental Figure S5 [file 41375_2021_1376_MOESM6_ESM.pdf]

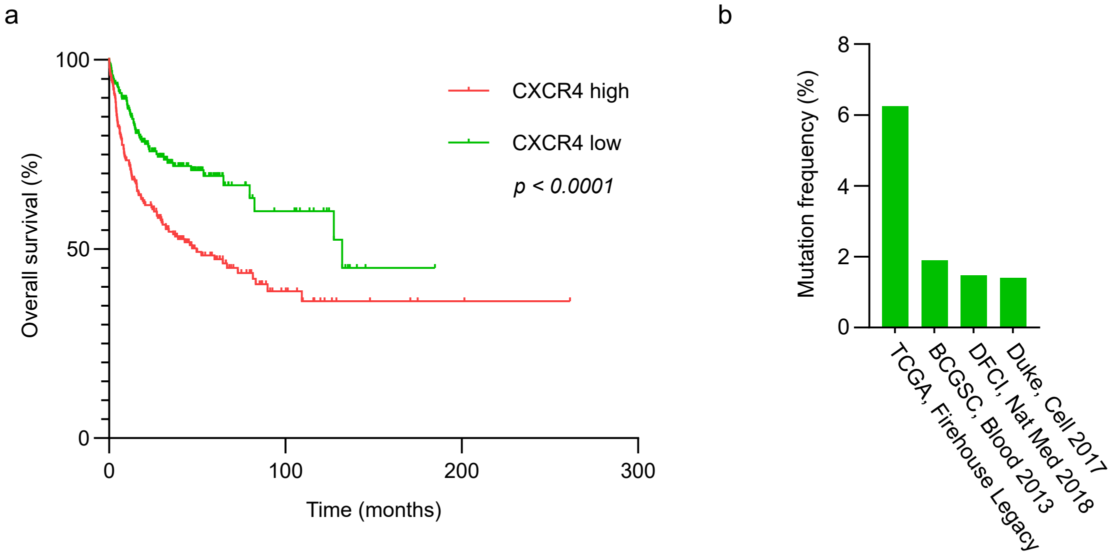

Supplemental Figure S5. Association of CXCR4 expression with survival and frequency of CXCR4 mutations in DLBCL patients.

(a) Kaplan-Meier survival analysis of 420 DLBCL Patients stratified by CXCR4 mRNA expression of GSE10846 (22), risk-group maximized and analyzed with SurvExpress (21). P value of log-rank (Mantel-Cox) test is shown. (b) Mutation frequency of CXCR4 in DLBCL patient datasets (TCGA, Firehose Legacy, n = 48; BCGSC, Blood 2013, n = 53; CDFCI, Nat Med 2018, n = 135; Duke, Cell 2017, n = 1001) (16, 17, 23).
