# Supplementary material for: CXCR4 hyperactivation cooperates with TCL1 in CLL development and aggressiveness
Source: Leukemia. 2021 Aug 6;35(10):2895–905. doi: 10.1038/s41375-021-01376-1 (PMC8478649; doi:10.1038/s41375-021-01376-1)
Supplement: Supplementary file 7 — Supplemental Figure S6 [file 41375_2021_1376_MOESM7_ESM.pdf]

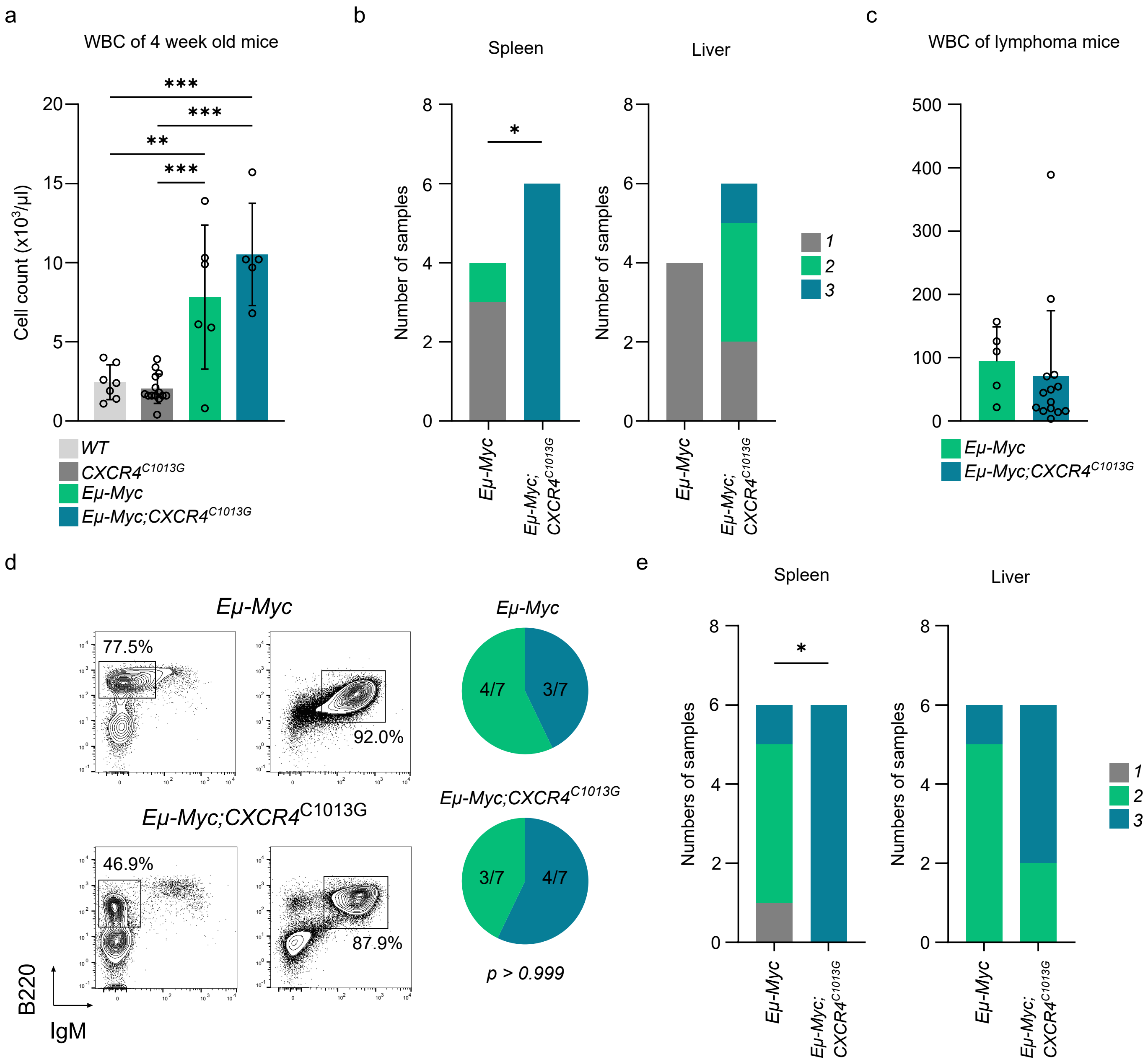

Supplemental Figure S6. Characterization of the *Eµ-Myc;CXCR4^{C1013G}* phenotype.

(a) White blood cell count (WBC) of 4 week old mice (*WT*, n = 7; *CXCR4^{C1013G}*, n = 14; *Eµ-Myc*, n = 6; *Eµ-Myc;CXCR4^{C1013G}*, n = 5).

(b) Quantification of histology scores (1-3) of spleen and liver from 4 week old mice (*Eµ-Myc*, n = 4; *Eµ-Myc;CXCR4^{C1013G}*, n = 6). Scorings system details are shown in Supplemental Methods.

(c) WBC of mice with manifest lymphoma (Eµ-Myc, n = 5; *Eµ-Myc;CXCR4^{C1013G}*, n = 14).

(d) Representative images of immunophenotyping of animals with manifest lymphoma by flow cytometry showing B220 and IgM expression of lymphoma cells (*Eµ-Myc*, n = 7; *Eµ-Myc;CXCR4^{C1013G}*, n = 7). Fractions of animals with either IgM+ or IgM- phenotype are indicated in the pie charts.

(e) Quantification of histology scores (1-3) of spleen and liver from mice presenting with manifest lymphoma (*Eµ-Myc*, n = 6; *Eµ-Myc;CXCR4^{C1013G}*, n = 6). Scorings system details are shown in Supplemental Methods.

Statistical analyses were performed with one-way ANOVA with Tukey correction for multiple comparisons (a), Student's t test (b), Mann-Whitney *U* test (c) and Fisher's exact test (d), * $P < 0.05$, ** $P < 0.01$, *** $P < 0.001$. Error bars indicate standard deviation (SD).
