# Supplementary material for: CXCR4 hyperactivation cooperates with TCL1 in CLL development and aggressiveness
Source: Leukemia. 2021 Aug 6;35(10):2895–905. doi: 10.1038/s41375-021-01376-1 (PMC8478649; doi:10.1038/s41375-021-01376-1)
Supplement: Supplementary file 8 — Supplemental Figure S7 [file 41375_2021_1376_MOESM8_ESM.pdf]

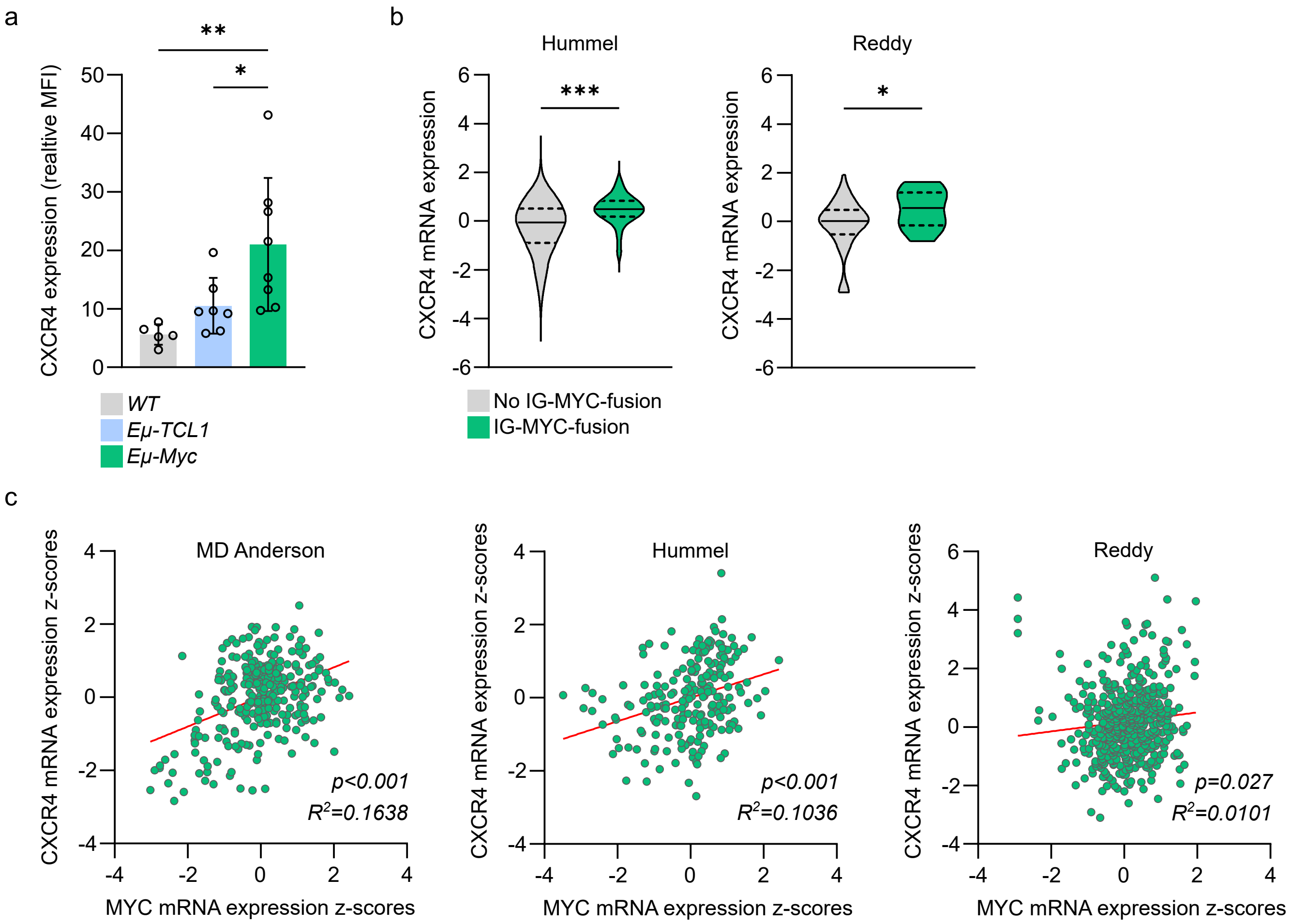

Supplemental Figure S7. CXCR4 surface and transcript expression is regulated by MYC.
(a) CXCR4 expression of tumors (*Eμ-Myc*, n = 8; *Eμ-TCL1*, n = 7) compared with healthy B cells from adult *WT* mice (n = 5), relative to isotype by flow cytometry.
(b) Correlation of CXCR4 mRNA expression dependent of IG-MYC fusion status from the Hummel data set (GSE4475) (24) (IG-MYC-fusion, n = 59; no IG-MYC-fusion n = 141, samples not annotated and samples with MYC translocations other than IG excluded) and the Reddy data set (16) (IG-MYC-fusion, n = 12; no IG-MYC-fusion, n= 80).
(c) Correlation of MYC mRNA and CXCR4 mRNA expression as log Z-scores relative to all samples in the MD Anderson Cancer Center data set (25), analyzed with cBioPortal (26, 27) (n = 286), the Hummel data set (GSE4475) (24) (n = 221) and the Reddy data set (16) (n = 482).
Statistical analyses were performed with one-way ANOVA with Tukey correction for multiple comparisons (a), Student's t-test (b) and Pearson correlation (c), * P < 0.05, ** P < 0.01, *** P < 0.001. Error bars indicate standard deviation (SD).
