# Supplementary material for: CXCR4 hyperactivation cooperates with TCL1 in CLL development and aggressiveness
Source: Leukemia. 2021 Aug 6;35(10):2895–905. doi: 10.1038/s41375-021-01376-1 (PMC8478649; doi:10.1038/s41375-021-01376-1)
Supplement: Supplementary file 9 — Supplemental Figure S8 [file 41375_2021_1376_MOESM9_ESM.pdf]

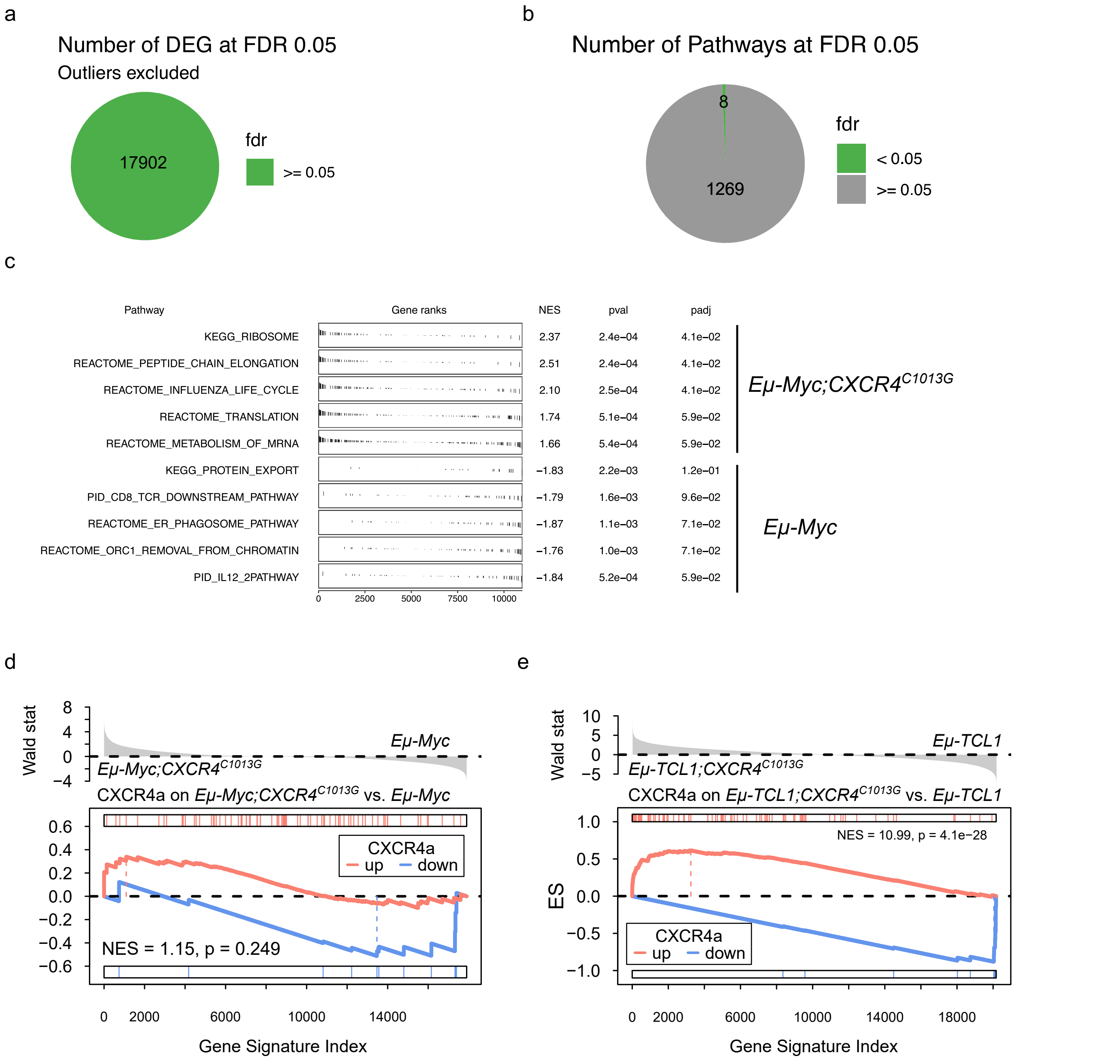

Supplemental Figure S8. RNA sequencing of *Eμ-Myc* and *Eμ-Myc;CXCR4^{C1013G}* lymphoma cells.
(a) DEG in *Eμ-Myc* (n = 6) vs. *Eμ-Myc;CXCR4^{C1013G}* (n = 6).
(b) GSEA for curated gene sets listed in MsigDB (7, 8) in *Eμ-Myc* vs. *Eμ-Myc;CXCR4^{C1013G}*.
(c) GSEA for curated gene sets listed in MsigDB (7, 8) in *Eμ-Myc* vs. *Eμ-Myc;CXCR4^{C1013G}* CD19+ B cells showing normalized enrichment scores (NES) P value(pval) and adjusted P value (padj).
(d) GSEA showing CXCR4a enrichment in *Eμ-Myc;CXCR4^{C1013G}* vs. *Eμ-Myc*.
(e) GSEA showing CXCR4a enrichment in *Eμ-TCL1;CXCR4^{C1013G}* vs. *Eμ-TCL1*.
