# Supplementary material for: CXCR4 hyperactivation cooperates with TCL1 in CLL development and aggressiveness
Source: Leukemia. 2021 Aug 6;35(10):2895–905. doi: 10.1038/s41375-021-01376-1 (PMC8478649; doi:10.1038/s41375-021-01376-1)
Supplement: Supplementary file 10 — Supplemental Figure S9 [file 41375_2021_1376_MOESM10_ESM.pdf]

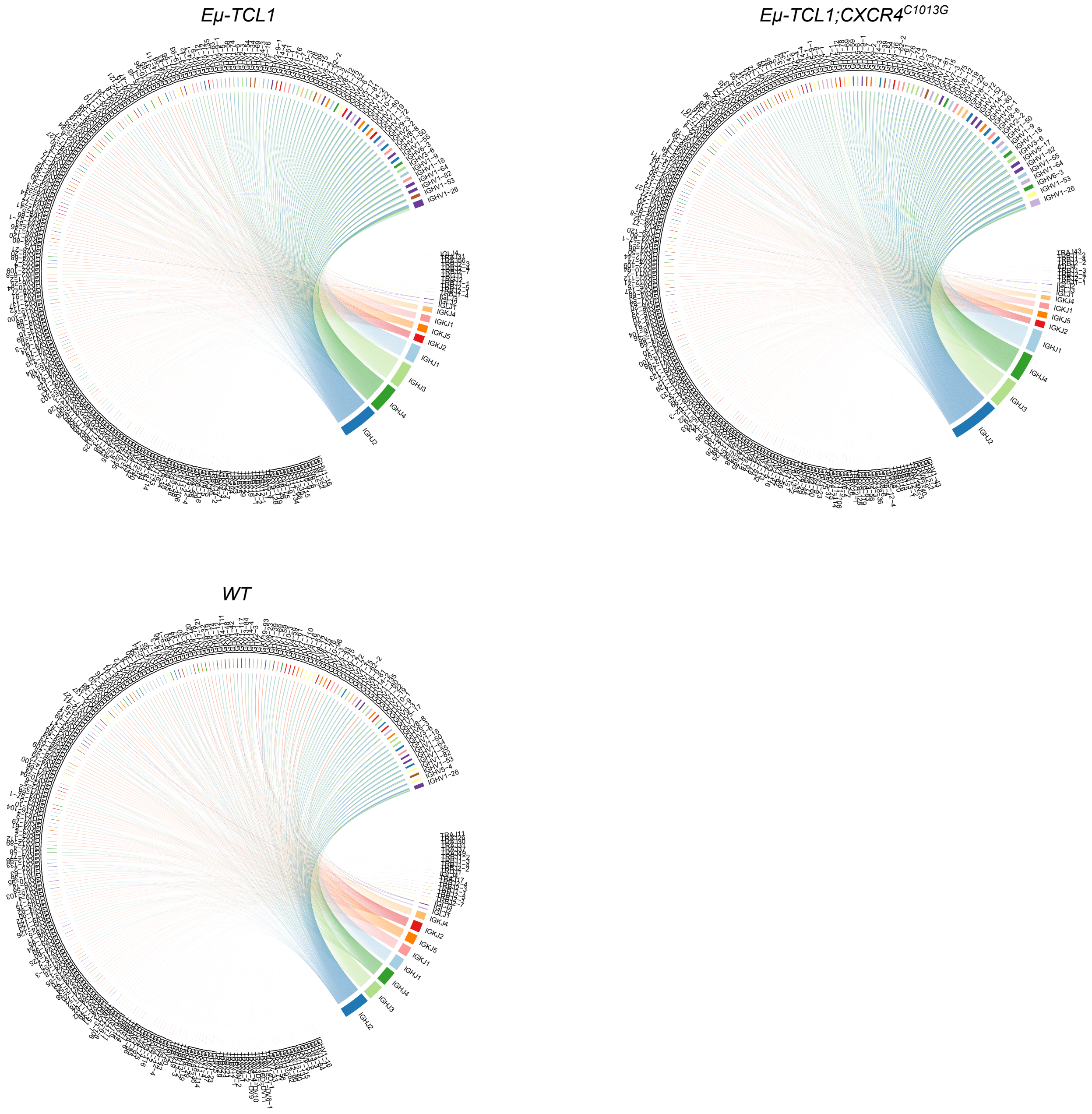

Supplemental Figure S9. Transcriptomic clonality analysis of CD19+ B cells in 6 week old mice.
Representative circos plots of V(D)J-rearrangement of CD19+ B cells of 6 week old mice (*Eμ-TCL1*, n = 5; *Eμ-TCL1;CXCR4*^{C1013G}, n = 5; *WT*, n = 5).
