# Supplementary material for: CXCR4 hyperactivation cooperates with TCL1 in CLL development and aggressiveness
Source: Leukemia. 2021 Aug 6;35(10):2895–905. doi: 10.1038/s41375-021-01376-1 (PMC8478649; doi:10.1038/s41375-021-01376-1)
Supplement: Supplementary file 11 — Supplemental Figure S10 [file 41375_2021_1376_MOESM11_ESM.pdf]

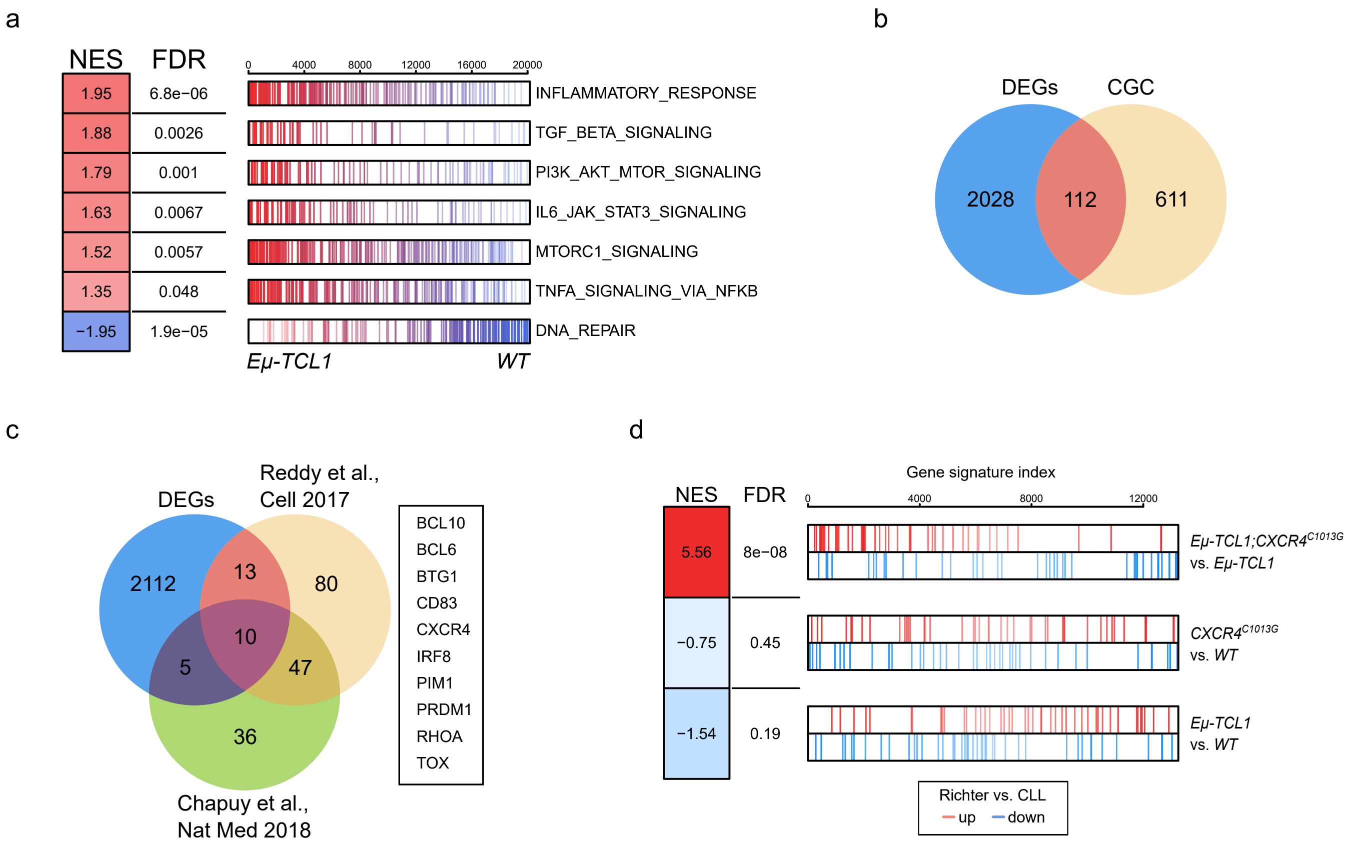

Supplemental Figure S10. Investigating the transcriptomic program of B cells from *Eμ-TCL1;CXCR4^{C1013G}* mice.

(a) GSEA of *Eμ-TCL1* vs. *WT* CD19+ B cells showing normalized enrichment scores (NES) and false discovery rates (FDR) for curated gene sets listed in MsigDB (7, 8).

(b) Overlap of DEG with padj < 0.05 of *Eμ-TCL1;CXCR4^{C1013G}* vs. *Eμ-TCL1* CD19+ B cells with the Cancer Gene Census (CGC) (15).

(c) Overlap of DEG with padj < 0.05 of *Eμ-TCL1;CXCR4^{C1013G}* vs. *Eμ-TCL1* CD19+ B cells with DLBCL drivers in patient datasets (16, 17).

(d) GSEA showing enrichment of a Richter's transformation signature in *Eμ-TCL1;CXCR4^{C1013G}* vs. *WT*, *Eμ-TCL1* vs. *WT* and *CXCR4^{C1013G}* vs. *WT* CD19+ B cells showing normalized enrichment scores (NES) and false discovery rates (FDR).
